# 35 Individuals With HUWE1-Related Neurodevelopmental Disorder and Suggested Clinical Evaluations

**DOI:** 10.1002/ajmg.a.70127

**Published:** 2026-04-13

**Authors:** Mindy H. Li, Deziree L. Coleman, Kelsey Hogan, Danielle Luz, Lindsay Bhandari, Newell Belnap, Tiffany Busa, Charles Coutton, Klaus Dieterich, Svetlana Gorokhova, Clara Hildebrandt, Rachel Logan, Milena Mariani, Manuela Morleo, Vincenzo Nigro, John Pappas, Rachel Rabin, Kelly Schoch, Angelo Selicorni, Vandana Shashi, Rebecca Spillmann, Jennifer Sullivan, Charlotte Tardy, Samantha A. Schrier Vergano, Brock Grill, Kristin Baranano

**Affiliations:** 1 Division of Genetics, Department of Pediatrics, Rush University Medical Center, Chicago, Illinois, USA; 2 Department of Pediatrics, Department of Pharmacology, University of Washington School of Medicine, Seattle, Washington, USA; 3 Norcliffe Foundation Center for Integrative Brain Research, Seattle Children's Research Institute, Seattle, Washington, USA; 4 Division of Pediatric Neurology, Department of Neurology, Johns Hopkins University School of Medicine, Baltimore, Maryland, USA; 5 Neurogenomics Division, Center for Rare Childhood Disorders, Translational Genomics Research Institute, Phoenix, Arizona, USA; 6 Department of Medical Genetics, Timone Children's Hospital, AP-HM, Marseille, France; 7 University Grenoble Alpes, Inserm, U1209, CHU Grenoble Alpes, Institut for Advanced Biosciences, Grenoble, France; 8 Aix Marseille Univ, INSERM, MMG, U 1251, Marseille, France; 9 Division of Genetics and Metabolism, Department of Pediatrics, University of North Carolina, Chapel Hill, Chapel Hill, North Carolina, USA; 10 Division of Neurosciences, Children's Healthcare of Atlanta, Atlanta, Georgia, USA; 11 Department of Pediatrics, ASST Lariana Sant'anna Hospital, Como, Italy; 12 Telethon Institute of Genetics and Medicine (TIGEM), Naples, Italy; 13 Department of Precision Medicine, University of Campania “Luigi Vanvitelli”, Naples, Italy; 14 Department of Pediatrics, Clinical Genetics Services, New York University Grossman School of Medicine, New York, New York, USA; 15 Division of Medical Genetics, Department of Pediatrics, Duke University School of Medicine, Durham, North Carolina, USA; 16 Department of Pediatrics, Eastern Virginia Medical School, Children's Hospital of the King's Daughters, Norfolk, Virginia, USA

## Abstract

*HUWE1* (HECT, UBA, and WWE Domain Containing E3 Ubiquitin Protein Ligase1, OMIM 300697), located at Xp11.22, encodes a ubiquitin ligase that is highly conserved across species. Genetic variants in *HUWE1* described in multiple independent studies cause X-linked intellectual disability, including in the patients identified by Juberg, Marsidi, and Brooks. This report describes 35 additional cases of individuals with variants in *HUWE1* and suggested guidelines for clinical management. Our study includes several female cases, which have not been widely reported previously. Our findings confirm earlier reported clinical features including developmental delay, autism, hypotonia, short stature, and dysmorphic facial features as well as additional multisystemic findings. Intrauterine growth restriction (IUGR) and feeding difficulties were common in the neonatal period. It is notable that nearly all females had *de novo* variants, and males had *de novo* or inherited variants from clinically unaffected carrier mothers. Three genetic hotspots were identified in evolutionarily conserved regions of HUWE1 that have clinical impact. This report provides additional characterization of the spectrum of HUWE1-related neurodevelopmental disorder (HNDD).

## 1 | Introduction

*HUWE1* (HECT, UBA, And WWE Domain Containing E3 Ubiquitin Protein Ligase1, OMIM 300697), located at Xp11.22, encodes a ubiquitin ligase that is highly conserved across species (Giles and Grill 2020; Grabarczyk et al. 2021; Huibregtse et al. 1995; Hunkeler et al. 2021). Multiple independent studies have identified variants in *HUWE1* across multiple patients that cause X-linked intellectual disability (XLID). This includes Juberg and Marsidi (1980) and Brooks et al. (1994) who reported individuals with XLID (OMIM 309590) that were subsequently found to have the same hemizygous variant (Gly4310Arg) (Friez et al. 2016). Froyen et al. (2008), Froyen et al. (2012) reported individuals with XLID with microduplications of Xp11.22 that included *HUWE1*, which appears to cause a distinct clinical syndrome. Additional studies have identified further individuals with *HUWE1* variants with common clinical findings including intellectual disability, seizures, short stature, and dysmorphic facial features (Froyen et al. 2008; Moortgat et al. 2018).

The role of HUWE1 in the nervous system remains relatively understudied compared with other genes associated with neurodevelopmental disorders. To date, findings from organismal models such as mice and *C. elegans* have shown that HUWE1 regulates early neural progenitor development, inhibitory GABAergic and glycinergic neuron function, and dendrite development (de Groot et al. 2014; Opperman et al. 2017; Urbán et al. 2016; Xie et al. 2023; Zhang et al. 2019; Zhao et al. 2008). HUWE1 was initially found to regulate neural progenitor development via ubiquitination of the MYC and Atoh1 transcription factors, as well as through effects on Wnt signaling (de Groot et al. 2014; Forget et al. 2014; Vandewalle et al. 2013; Zhao et al. 2009). Recent studies with human cerebral organoids demonstrated HUWE1 effects on neural progenitor development could occur through p53 (Aprigliano et al. 2021). HUWE1 regulation of GABA neuron function occurs via both ubiquitin ligase activity and protein complex formation with the OGT-1 glycosyltransferase (Giles et al. 2019). Effects on glycinergic transmission occur by HUWE1 ubiquitination and trafficking effects on the glycine receptor GlyRα1 (Zhang et al. 2019).

Although more than 30 individuals have been described with disorder-associated variants in *HUWE1*, there is limited long-term data on the outcomes of these individuals. There are also no established clinical guidelines or treatment for *HUWE1* developmental and neurobehavioral abnormalities. Here, we present 35 additional individuals with variants in *HUWE1* that present with developmental delay and XLID. While many variants are of unknown pathological significance, several are present in multiple patients from our study or prior studies indicating they are likely to be causal (Friez et al. 2016; Froyen et al. 2008; Moortgat et al. 2018). Five additional probands from two families were recently described (De Falco et al. 2025), and this group proposed to refer to the neurobehavioral spectrum disorder related to HUWE1 as *HUWE1*-related neurodevelopmental disorder, which we refer to as HNDD.

## 2 | Materials and Methods

### 2.1 | Patient Identification

Approval from the Institutional Review Boards at Rush University Medical Center (#199022204-IRB01) and Johns Hopkins University Medical Center (IRB00256078) was obtained and appropriate informed written consents. 35 individuals with XLID and variants in *HUWE1* were identified during genetic workup for developmental delay and/or other medical issues. Clinical genetic testing was completed at certified laboratories that followed standard guidelines for variant interpretation. A letter was provided to GeneDx to connect the authors with interested ordering physicians and researchers who had patients identified with variants in *HUWE1* in that laboratory. A few patients were also connected to the study through GeneMatcher (Sobreira et al. 2015). Consent from families was obtained for participation in this case series. Families provided additional photo consent if they consented to submitting facial photographs. For patients enrolled through Rush University Medical Center, clinical care providers retrospectively reviewed and summarized available medical and phenotypic information. For patients enrolled through Johns Hopkins University, clinical information was obtained through a structured clinical interview performed by the same clinician, either in person or via Zoom. Data were manually summarized and reviewed for analysis. All variants were either confirmed to have been previously submitted to ClinVar or were deposited.

### 2.2 | HUWE1 Protein Sequence Alignment and Analysis

HUWE1 protein sequences from different species were aligned and evaluated for evolutionary conservation using the Clustal Omega (1.2.4) multiple sequence alignment tool. HUWE1 protein sequences used for alignments were derived from reference mRNA sequences for human HUWE1 (NM_031407.7), mouse HUWE1 (NM_021523.5), zebrafish HUWE1 (NM_001365654.1), *Drosophila* HUWE1 (NM_132831.2), and *C. elegans* EEL-1/HUWE1 (NM_067883.5). Amino acid residues in HUWE1 where patient-associated missense variants occur were manually curated and annotated in sequence alignments.

## 3 | Results

### 3.1 | Patient Findings

#### 3.1.1 | Patient Demographics

A total of 35 individuals from 32 families were identified, 22 males and 13 females (summarized in Table 1). Sex was assigned based on clinician report. The ages ranged from 18 months to 52 years. The majority were referred due to concern for developmental delays and/or other significant medical concerns such as intrauterine growth restriction (IUGR), failure to thrive (FTT), or seizures. All *HUWE1* variants in females were *de novo*, with one exception where her father had suspected mosaicism. This is based on the presence of the variant in a small percentage of the reads from the father's sample from trio whole exome sequencing. For the affected males, 11 had *de novo* variants in *HUWE1*. For 8 families, the variant was inherited. One family has described 3 affected males, and another has described 2 affected males. Uniformly, the carrier mothers were described as normal, meaning no history of intellectual disability, seizures, or other neurodevelopmental concerns. X-inactivation was reported only for one mother; in this case (patient 8), it was skewed at 93:7. Additional variants of potential clinical significance were identified in two individuals (in *TUBB3* for Patient 30 and *SCN2A* for Patient 32) that likely contributed to the reported features of developmental delay, hearing loss, and/or seizures. However, these disorders are not generally thought to have manifestations outside of the nervous system, and given the relatively common finding of dual diagnoses, estimated at 5% (Spedicati et al. 2022), we chose to include them in the analysis.

#### 3.1.2 | Neurological

Developmental delays were a core clinical feature. Motor delays were reported in 20/22 (91%) males and 11/13 (85%) females (Table 2; Table S1). Hypotonia was present in 17/22 (77%) males and 8/13 (62%) females. 16/22 (73%) males had achieved the ability to walk, at the average age of 29 months (range 15 months to 8 years). For the 6 who had not, 3 of them were able to sit without support. 11/13 (85%) females walked, at an average age of 25 months (range 12 months to 3 years). One individual was only 18 months at the time of the report.

Language delays were nearly universally reported, in 22/22 (100%) of males and in 12/13 (92%) of females. 12/22 (55%) males were nonverbal, compared with 3/13 (23%) females. Clinicians did not reliably report if intellectual disability (ID) was present or the degree of severity. In some cases, that was a function of the age of the child; hence they used instead the term global developmental delay (GDD). When this information was provided, males were typically described in the range of moderate to severe GDD/ID, whereas females were mild to moderate.

Autism spectrum disorder or significant autistic features were diagnosed with some frequency, 9/22 (41%) for males and 4/13 (31%) for females.

Seizures were frequently an issue, reported for 9/22 (41%) of males and 4/13 (31%) of females. Seizures were typically relatively easy to control, typically with monotherapy for the first or second trialed antiseizure medication, with three notable exceptions: the individual with an additional *SCN2A* variant, for 3 individuals with *de novo* variants at Arg3070His/Cys (Patients 10–12), and for one individual with Arg3998Cys. For the latter two locations, their epilepsy was described as pharmaco-resistant.

Nearly all individuals had undergone MRI of the brain, as part of the evaluation of hypotonia, GDD, and/or seizures. An Arnold-Chiari malformation was reported in 3 individuals, all with pathogenic *de novo* Arg110Gln/Trp variants. White matter volume loss was reported in a number of cases (*n* = 6). Two cases had serial imaging that demonstrated progressive white matter volume loss with time, one of which had associated intractable epilepsy (Patients 10 and 12).

#### 3.1.3 | Prenatal and Postnatal Growth

Intrauterine growth restriction (IUGR) was reported for 7/21 males (33%) and 2/13 (15%) females. Postnatally, short stature was a frequent concern, described in 16/22 males (73%) and 8/13 (62%) of females, although not reliably less than the 3^rd^ percentile for height. In general, patients who were evaluated by endocrinology were not found to be deficient in growth hormone. At least two patients (Patients 16 and 22) anecdotally had a robust response to growth hormone therapy after it was approved after a failed growth hormone stimulation test. Microcephaly, as defined by head circumference less than the third percentile, was described in 3 females and 5 males.

#### 3.1.4 | Facial Characteristics

**FIGURE 1 |:**
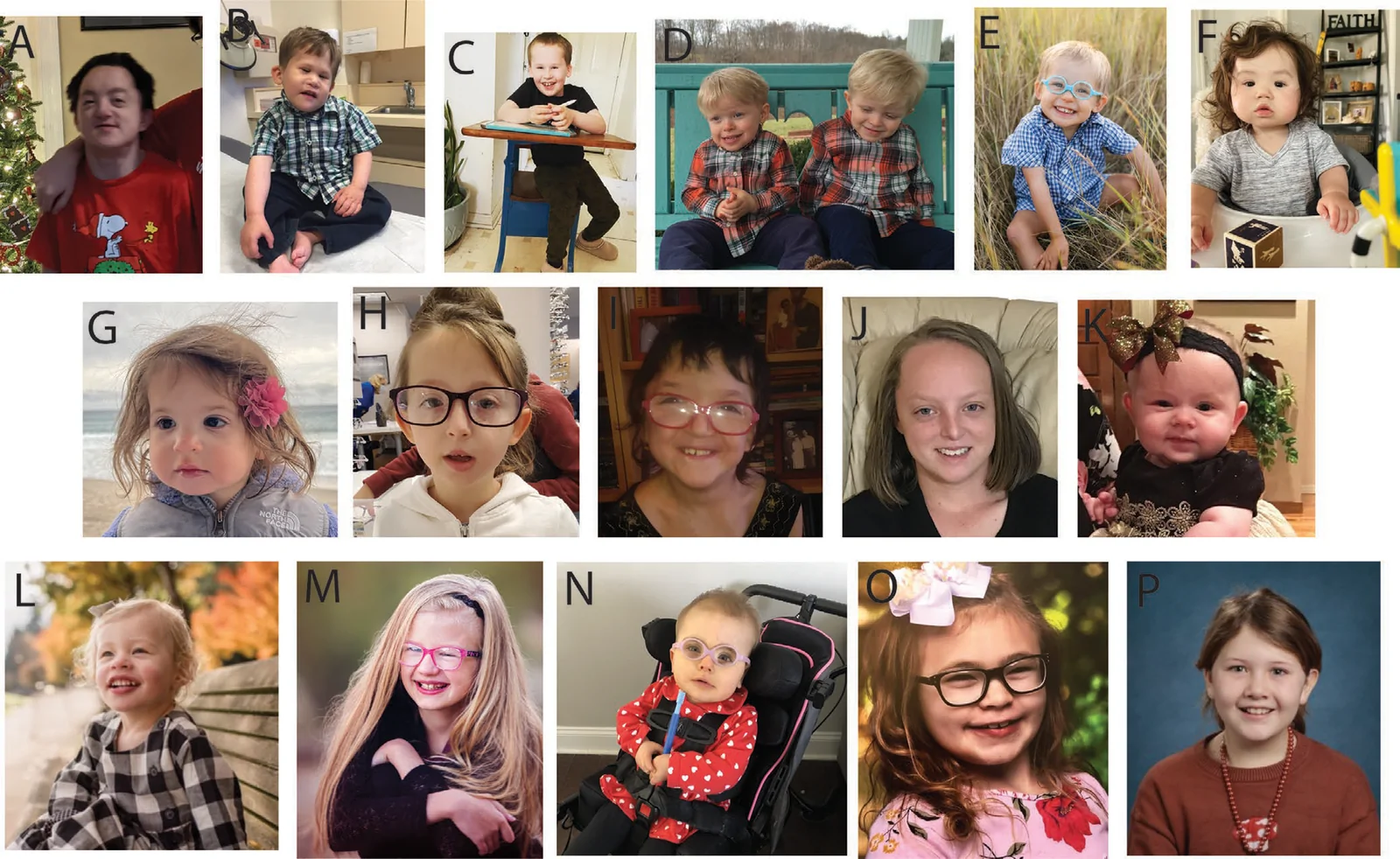
Representative patient photos of individuals with *HUWE1* variants. Males (A–F), females (G–P). (A): Patient 2, Arg110Trp; (B): Patient 11, Arg3070His; (C): Patient 10, Arg3070Cys; (D): Patient 16, mosaic for Thr3717Ala, with his twin brother; (E): Patient 18, Gln3698Pro; (F): Patient 22, Gly4310Arg; (G): Patient 23, Glu50Lys; (H): Patient 24, Glu105Val; (I): Patient 26, Arg110Gln; (J): Patient 27, Arg110Trp; (K): Patient 28, c.646–1G > A; (L): Patient 29, Met1328Val; (M): Patient 30, Lys2356Phe; (N): Patient 33, Arg3998Cys; (O): Patient 34, Gly4229Asp; (P): Patient 35, Gly4229Arg. Commonly observed features include deep set eyes with epicanthal folds.

Facial dysmorphisms were described by the reporting clinician. For individuals evaluated via video, examination was frequently limited by the quality of the connection and/or patient cooperation. In such cases, family-provided photographs supplemented the evaluation. Family-provided photographs are presented in Figure 1. Complete descriptions are available in Table S1. In general for males, some had a recognizable facial appearance. Common features include hypotelorism, almond-shaped palpebral fissures, and epicanthal folds.

#### 3.1.5 | Vision

Ophthalmological concerns were common. Abnormal vision was frequently reported in 14/22 (54%) males and 5/13 (38%) females. This was typically refraction errors, but one boy was described as being blind. Strabismus was similarly reported in 15/22 males (68%) and 6/13 (46%) females.

#### 3.1.6 | Hearing

Hearing loss was found in 4/22 (18%) males and 4/12 (33%) females. Hearing aids were reported for 2 individuals and cochlear implant surgery for one. Hearing loss was not described as being progressive.

#### 3.1.7 | Dental

Dental issues were reported in 6/22 (27%) males and 9/13 (69%) females. These concerns ranged from dental caries to small and missing adult teeth.

#### 3.1.8 | Feeding Problems

Feeding difficulties during infancy were common. They were reported in 14/22 (63%) males and 6/13 (46%) females. Feeding tubes were required for 9 males and 1 female. Feeding issues frequently improved with time, reported at the current age in 8/22 (36%) males and 2/13 (15%) females.

#### 3.1.9 | Cardiovascular Concerns

Congenital heart defects were reported in 7/22 (32%) males and 2/13 (15%) females. These included patent foramen ovale (*n* = 2), patent ductus arteriosus (*n* = 3), atrial septal defect (*n* = 3), and one case of a complex heart defect including atrial and ventricular septal defects, ventricular hypoplasia, and tricuspid atresia.

#### 3.1.10 | Skeletal and Orthopedic Issues

Craniosynostosis was found in 3 individuals with *de novo* variants Arg110Gln/Trp (1 male, 2 females). Scoliosis was a concern for 4/13 (31%) females and 5/22 (23%) males. Two males required hip osteotomies.

Major clinical features with associated frequencies are summarized in table 2 where our findings are compared with the cohort from Moortgat et al. (2018) with 21 individuals (7 male, 14 female). Our data is more granular but generally aligns with their findings in males versus females. Please note that Moortgat et al. (2018) did not specifically report on prenatal IUGR, congenital heart defects, or the need for a feeding tube. Their reporting was relatively incomplete for autistic features and for seizures. Complete patient phenotype reporting is provided in Table S1.

### 3.2 | Sequence and Evolutionary Analysis of Clinical Variants

#### 3.2.1 | HUWE1 Missense Variants Define Three Genetic Hotspots and Affect Evolutionarily Conserved Residues

Having identified numerous patients with genetic variants in *HUWE1* associated with neurobehavioral abnormalities, bioinformatic analysis was used to further evaluate these genetic changes. Analysis focused on two primary goals. (1) Mapping where missense variants occur in the HUWE1 protein sequence, and (2) examining whether individual genetic changes occur at residues that are evolutionarily conserved.

**FIGURE 2 |:**
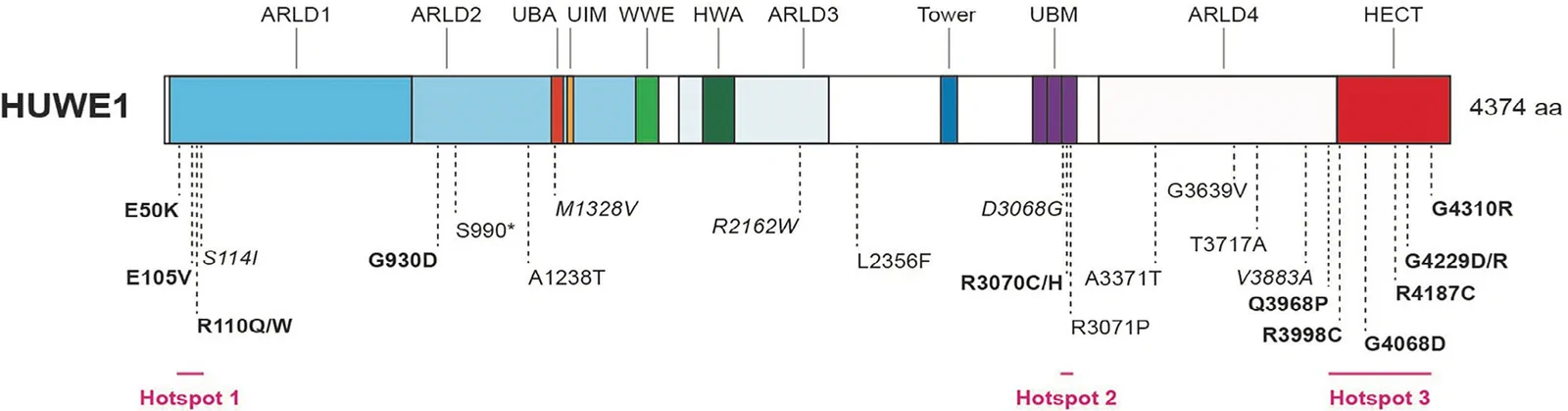
*HUWE1* genetic variants identified in patients highlight three genetic hotspots. Schematic of human HUWE1 protein sequence with annotation of patient variants. Variants were identified broadly across HUWE1 sequence. Three genetic hotspots where multiple variants occurred in close proximity in linear protein sequence were identified in the N-terminal region of ARLD1 (hotspot 1), in the UBM domain (hotspot 2), and the enzymatic HECT ubiquitin ligase domain (hotspot 3). Residues where patient variants occurred were evaluated for evolutionary conservation between human HUWE1 and *C. elegans* EEL-1/HUWE1. Highlighted are variants that affect identical residues (bold) or conserved residues (italics). Note genetic variants identified in females and males did not concentrate in hotspots and are widely distributed across HUWE1 sequence for both sexes.

HUWE1 is a HECT family ubiquitin ligase with broad functions in nervous system development (Giles and Grill 2020). Owing to its immense physical size (over 400 kDa), HUWE1 contains numerous conserved protein domains many that lack a known function (Figure 2). Structural and biochemical studies on HUWE1 have identified multiple regions of the protein involved in ubiquitin ligase activity (Grabarczyk et al. 2021; Hunkeler et al. 2021; Pandya et al. 2010). The primary enzymatic ubiquitin ligase domain is the HECT domain, which catalyzes conjugation of ubiquitin to substrates. The ubiquitin binding motif (UBM) repeat domain is predicted to bind ubiquitin. Finally, recent studies have suggested that a large N-terminal portion of HUWE1 that contains 4 armadillo repeat-like domains (ARLDs), a ubiquitin associated (UBA) domain and a ubiquitin interacting motif (UIM) also influences ubiquitin ligase activity (Hunkeler et al. 2021; Walinda et al. 2014).

Bioinformatic analysis indicated that the 34 patient missense variants in *HUWE1* reported affect 23 amino acid residues, with variants occurring broadly across the HUWE1 protein sequence (Figure 2 and Table 1; Figure S1). The 35 total patients identified also include an individual with a splice site variant (patient 16) that was not analyzed further. To our knowledge, this study also identified the first patient (male) with a premature stop (Ser990*), which would potentially result in a truncated HUWE1 protein. All residues where missense variants occur were evaluated for amino acid conservation across five species spanning human HUWE1 through *C. elegans* EEL-1/HUWE1. Results indicate that patient variants occur at 11 residues that are identical across species, 5 residues that are conserved, and 7 residues that are not conserved (Figure 2, Figure S1). Notably, missense changes that affect conserved residues occur in patients carrying either *de novo* (15/24 patients) or inherited (7/11) missense *HUWE1* variants.

Interestingly, the patient cohort revealed three genetic hotspots in *HUWE1* where multiple variants occur in close proximity in the linear protein sequence (Figure 2, Figure S1). The first hotspot occurs in an N-terminal region of ARLD1 between Glu50 and Ser114 where five variants occur (Glu50Lys, Glu105Val, Arg110Gln, Arg110Trp and Ser114Ile). Variants in hotspot 1 were identified in 8 patients; all are *de novo* monoallelic lesions, and affect highly conserved residues (Figure 2, Figure S1, Table S1). Hotspot two is in a very small protein motif between Asp3068 and Arg3071 in the third repeat of the UBM domain (Figure 2, Figure S1). There are four variants located in hot spot 2 (Asp3068Gly, Arg3070His, Arg3070Cys and Arg3071Pro) that were present in 6 patients (Table S1). Variants in hot spot 2 display variable inheritance and affect residues with varying degrees of conservation (Figure 2, Figure S1, Table S1). Finally, hotspot 3 contained variants in or near the catalytic HECT domain between Gln3968 and Gly4310 which harbors 7 variants (Gln3968Pro, Arg3998Cys, Gly4068Asp, Arg4187Cys, Gly4229Asp, Gly4229Arg and Gly4310Arg) that were identified in 7 patients and all occur at highly conserved residues (Figure 2, Figure S1, Table S1). Genetic variants identified in females and males did not concentrate in any of the three hotspots identified.

Recurrent variants were observed at Glu50 (*n* = 2 patients), Arg110 (*n* = 4), Arg3070 (*n* = 4), and Gly4229 (*n* = 2). The patients with Glu50Lys, Arg110Gln/Trp, and Gly4229Asp/Arg variants have *de novo* lesions that affect highly conserved residues (Figure 2, Figure S1, Table S1). There are three patients with the inherited variant Gly930Asp who are members of the same family, as are the two patients with the inherited variant Arg4187Cys.

Thus, bioinformatic analysis identified numerous *HUWE1* variants that affect residues that are evolutionarily conserved from *C. elegans* through humans. This suggests such variants are likely to be functionally or structurally important. The patient cohort here reinforces evidence for genetic hotspots 1 and 3, which were previously noted for *HUWE1* variants associated with non-syndromic and syndromic intellectual disability (Giles and Grill 2020). Interestingly, hotspot 2 was revealed for the first time in the patient population profiled here.

## 4 | Discussion

Despite the substantial evidence for HNDD as a distinct clinical entity, HUWE1 remains relatively understudied in the nervous system. This study describes the largest cohort of HUWE1 patients to date. Multiple new variants were identified, which substantially expands the number of reported genetic variants in *HUWE1* associated with HNDD. Extensive clinical profiling supports the concept that intellectual disability and/or developmental delay is a hallmark of HNDD. However, it is important to note that patients with HNDD display a spectrum of neurodevelopmental abnormalities with varying severity of intellectual disability and developmental delay, seizure, reduced motor coordination, impaired speech, hypotonia and autism. Both *de novo* and maternally inherited changes were found in males, but no inherited variants were found in affected females, with the exception of a mosaic variant inherited from a father (Patient 31). There was no clear clinical correlation between variant location or evolutionary conservation and the severity of intellectual disability or developmental delays. The four individuals with variants at the Arg3070 residue had a consistent phenotype including the core features of intellectual disability, distinct facial features, short stature and epilepsy. There were two unrelated girls with Gly4229Asp (Patient 34) and Gly4229Arg (Patient 35) variants who were discordant in cognitive abilities (moderate intellectual disability versus normal intelligence, respectively), but it is unclear if this might reflect variable skewing of X-inactivation, variable penetrance, or differing effects of the amino acid substitution. In this situation, a nonpolar glycine is replaced by either a polar, negatively charged aspartic acid or a polar, positively charged arginine. The Gly4229Arg individual underwent genetic evaluation due to features of autism spectrum disorder, psychiatric symptoms resistant to medication treatment and short stature.

The patient population featured several recurrent variants that affected the same residue, and are likely to be causal for HNDD. This included variants identified in multiple patients, such as Arg3070Cys/His and Glu50Lys. There was also evidence for recurrent variants from prior studies identified in further new patients here such as: (1) Gly4310Arg associated with Juberg-Marsidi Brooks syndrome and identified by Friez et al. (2016); (2) Arg110Gln/Trp (Moortgat et al. 2018); (3) Arg3070Cys (Moortgat et al. 2018); (4) Arg4187Cys (Froyen et al. 2008); and (5) Met1328Val (Moortgat et al. 2018). One variant, Ser114Ile, was directly adjacent to Ser115Phe, which was previously identified (Moortgat et al. 2018). The cohort here also revealed numerous novel variants that were only identified in a single patient. At present, the functional genetic impact of *HUWE1* genetic variants remains largely unknown. A prior structural biochemical study demonstrated that Arg4187Cys (identified again here) shows a slight impairment in E3 ligase activity (Hunkeler et al. 2021). Prior work in the *C. elegans* model showed two prior variants not identified in this study, Arg2991His and Arg3990Cys, reduce HUWE1/EEL-1 function (Opperman et al. 2017). Studies with human cerebral organoids have confirmed that the originally described *HUWE1* Gly4310Arg variant affects neural progenitor development (Aprigliano et al. 2021; Brooks et al. 1994; Juberg and Marsidi 1980). Thus, while little is known about how *HUWE1* variants affect gene function, it is possible variants identified here could result in loss of function. However, this will need to be determined with future experimental testing.

Importantly, HNDD affects multiple organ systems based on clinical data described in Table 2 and Table S1. Consistent with HNDD being a spectrum disorder, nearly all individuals were reported to have developmental delay, but there was a range of severity. In addition to severely affected males, there were also severely affected females consistent with what has been reported previously by Moortgat et al. (2018). While many of the manifestations of HNDD are common with XLID/NDD syndromes, given the degree of medical complexity that can occur for HNDD, we provide proposed clinical evaluation and management guidelines described in Table 3. These clinical guidelines will provide a starting point for clinical evaluation of future patients with a potential diagnosis of HNDD.

As anticipated with an X-linked condition, males were generally more severely affected by HNDD. Mouse models with conditional knock out of HUWE1 in the developing central nervous system are non-viable (D'Arca et al. 2010; Zhao et al. 2009). The one male individual reported with a truncating variant that presumably would result in loss of HUWE1 function is very profoundly affected. He has such severe growth restriction that an initial diagnosis of primordial dwarfism was considered. He is unable to sit without support, nonverbal and minimally interactive with the environment. One female with a splice site variant is reported. While X-inactivation studies were not performed, her facial features, especially the appearance of her eyes (Figure 1K) are consistent with HNDD. Figure 1 demonstrates the range of facial features with some overlapping facial characteristics. As additional individuals are identified, the facial morphology of this condition will continue to be refined.

Interestingly, craniosynostosis was only reported for individuals with variants in Arg110. This falls within the first identified hotspot, in the ARLD1 domain, but other variants in this hotspot are not associated with craniosynostosis. Pharmaco-resistant epilepsy was associated with variants at Arg3070, within the second identified hotspot in the UBM domain. As might be anticipated, the third hotspot is in and near the HECT domain, which conveys substrate specificity for the E3 ligase activity of HUWE1. The only variant associated with severe epilepsy in this third hotspot was Arg3998Cys.

Prior to the identification of HNDD, HUWE1 had primarily been studied for its role in cancer biology. A common theme in neurodevelopmental disorders is for developmentally important genes to be involved in tumorogenesis (Chen et al. 2015; Di Cristofano et al. 1998; Fernández-Martínez et al. 2015; Levy et al. 2021; van Bon et al. 2016). At present, it is unclear if children with HNDD will develop tumor susceptibility later in life. Ongoing natural history studies in aging patients will be crucial in answering questions such as this. Currently most of the adults in our cohort (4/5) are in their early 20s so potential susceptibility to cancer and other changes that may occur over time, such as facial features, remain to be determined. With our expanded cohort of patients and more extensive clinical profiling in place, a better understanding of the molecular genetic basis of HNDD now awaits future experimental genetic studies examining the functional effects of specific *HUWE1* variants.

## 5 | Limitations

Some information was self-reported by a parent/guardian and others were obtained through retrospective chart review by clinicians. Due to this, there was variation in reported clinical information for individuals. For patients who were evaluated by other specialties aside from medical genetics, there were fewer dysmorphology details available.

## 6 | Conclusions

In conclusion, this study presents the largest cohort of new cases of individuals with variants in *HUWE1* presenting with developmental delay, XLID, seizures and/or autism. Thus, we propose to refer to the neurobehavioral spectrum disorder related to HUWE1 as HUWE1-related neurodevelopmental disorder (HNDD). The number of new patients and variants described here substantially broadens the clinical phenotype of individuals with this disorder. There are also three specific genetic regions in *HUWE1* that have clinical implications. Long-term, natural history studies will be needed to determine clinical health status and risks for individuals with HNDD.

## Supplementary Material

Figure S1

Table S1

Supporting Information

Additional supporting information can be found online in the Supporting Information section. Figure S1: Protein sequence alignment for human HUWE1 through *C. elegans* EEL-1/HUWE1 demonstrates patient-associated variants affect evolutionarily conserved amino acid residues. Clustal Omega was used to align full-length HUWE1 protein sequences from multiple species. Shown are sequence alignments between HUWE1 from human (hHUWE1), mouse (mHUWE1), zebrafish (zHUWE1), *Drosophila* (dHUWE1), and *C. elegans* (cEEL-1). Annotated are identical residues (*) highly conserved residues (:) and mildly conserved residues (.) residues are highlighted by colored boxes to indicate variants observed in only females (red), only males (blue), or both females and males (purple). Table S1: Complete patient phenotype table.

## Figures and Tables

**TABLE 1 |:** Listing of families 1–32 with associated *HUWE1* variants.

Family	Patient variant

1	E50K
2	E50K
3	E105V
4	R110W
5	R110Q
6	R110Q
7	R110W
8	S114I
9	C.646-1G > A
10	G930D
10	G930D
10	G930D
11	S990*
12	A1238T
13	M1328V
14	R2162W
15	L2356F
16	D3068G
17	R3070C
18	R3070H
19	R3070C
20	R3070C
21	R3071P
22	A3371T
23	G3639V
24	T3717A
25	V3883A
26	Q3968P
27	R3998C
28	G4086D
29	R4187C
29	R4187C
30	G4229D
31	G4229R
32	G4310R

*Note:* Variants of males indicated in blue and females in red.

**TABLE 2 |:** Summary of frequency of major clinical features in individuals with *HUWE1* variants.

Clinical features	Males (*n* = 22)	Moortgat et al. (2018) males (*n* = 7)	Females (*n* = 13)	Moortgat et al. (2018) females (*n* = 14)

Craniosynostosis	1/22 (4.5%)	0/7 (0%)	2/13 (15%)	2/14 (14%)
Strabismus	15/22 (68%)	5/6 (83%)	6/13 (46%)	8/14 (57%)
Hearing loss	4/22 (18%)	2/6 (33%)	4/12 (33%)	2/12 (17%)
Prenatal IUGR	7/21 (33%)	NR	2/13 (15%)	NR
Short stature	16/22 (73%)	6/7 (86%)	8/13 (62%)	9/14 (64%)
Congenital heart defect	7/22 (32%)	NR	2/13 (15%)	NR
Need for feeding tube	9/22 (27%)	NR	1/13 (7.6%)	NR
Motor delays	20/22 (91%)	7/7 (100%)	11/13 (85%)	13/14 (93%)
Language delays	22/22 (100%)	6/7 (86%)	12/13 (92%)	13/14 (93%)
Hypotonia	17/22 (77%)	4/6 (67%)	8/13 (62%)	10/14 (71%)
ASD or autistic features	9/22 (41%)	2/3 (67%)	4/13 (31%)	5/8 (63%)
Seizures	9/22 (41%)	3/4 (75%)	4/13 (31%)	4/14 (29%)

*Note:* Percentages in males versus females are compared with those reported in Moortgat et al. (2018). NR indicates not recorded.

**TABLE 3 |:** Summary of suggested clinical management recommendations upon diagnosis of HUWE1-related neurodevelopmental disorder (HNDD).

System/concern	Evaluation	Comments

Constitutional	Measurement of height, weight and head circumference	
Neurologic	Neurological evaluation	To include brain MRI, EEG if concern for seizures
Development	Developmental assessment	Motor, adaptive, cognitive and speech/language evaluation; refer for early intervention services
Behavioral	Autism evaluation	If concern for autistic features
Musculoskeletal	Neurosurgical and/or orthopedic evaluations	If craniosynostosis, Chiari malformations, scoliosis or hip issues are present
Gastrointestinal/feeding	GI/nutrition/feeding team evaluations	If feeding issues are present; management of constipation
Cardiovascular	Echocardiogram	To rule of structural heart defect
Eyes	Ophthalmological evaluation	To assess for refraction errors and strabismus
Hearing	Audiologic evaluation	To assess for hearing loss; hearing aids and cochlear implants if appropriate
Dental	Dental evaluation	
Endocrine	Refer for evaluation of short stature	Consideration of growth hormone treatment

## Data Availability

The data that supports the findings of this study are available in the S1 of this article.
