# Supplementary material for: 35 Individuals With HUWE1-Related Neurodevelopmental Disorder and Suggested Clinical Evaluations
Source: Am J Med Genet A. Author manuscript; Available in PMC 2026 Sep 15. (PMC13577160; doi:10.1002/ajmg.a.70127)
Supplement: Supplementary file 1 [file NIHMS2204174-supplement-Figure_S1.docx]

Supplemental Figure 1

**E50K**

```
hHUWE1      MKVDRTKLKKT--PTEAPADCRALIDKLKVCNDEQ-LLLELQ--QIKTWNIGKCELYHWV	55
mHUWE1      MKVDRTKLKKT--PTEAPADCRALIDKLKVCNDEQ-LLLELQ--QIKTWNIGKCELYHWV	55
zHUWE1      MKVDRSKLKKT--PTEAPADCRTLIEKLKGCSDEQ-LLLELQ--QIKTWNIGKCELYHWV	55
dHUWE1      MKVDTVKLKKG--SSELTAECRELIDELTKRRTRAELLEFLD--TVHVWIYGKCELYHWI	56
cEEL-1      MKIDDAEPSSSSSGSDMPPASATLLRNIVATKNDEDFIAAINKGREVHAVMGKTELYKWT	60
            **:*  : ..    ::    .  *: ::        ::  ::         ** ***:*
```

Hotspot 1 start

ARLD1 start

**S114I**

**R110Q/W**

**E105V**

x

```
hHUWE1      DLLDRFDGILADAGQTVENMSWMLVCDRPEREQLKMLLLAVLNFTALLIEYSFSRHLYSS	115
mHUWE1      DLLDRFDGILADAGQTVENMSWMLVCDRPEKEQLKMLLLAVLNFTALLIEYSFSRHLYSS	115
zHUWE1      DLLDRFDGILCDAGQTVENMSWLLVCDRPENGQLKALLLAVLNFTALLIEYSFSRHLYSS	115
dHUWE1      EILDRFDKILEEASRHVNGNEFVLQCDTNFQETDVTLLLHVLNFTTLLIEHSFSRHLYNS	116
cEEL-1      EVLNRCDEILEKAVHKNEF--GNLQCDHDT--VLKNHAVAIVRFTVLLFECTSSRRIYKS	116
            ::*:* * ** .* :  :     * **           : ::.**.**:* : **::*.*
```

```
hHUWE1      IEHLTTLLASSDMQVVLAVLNLLYVFSKRSNYIT-RLGSDKRTPLLTRLQHLAESWGGKE	174
mHUWE1      IEHLTTLLASSDMQVVLAVLNLLYVFSKRSNYIT-RLGSDKRTPLLTRLQHLAESWGGKE	174
zHUWE1      IEHLTTLLASCDMQVVLSVLNLLYVFSKRSNYIT-RLGSDKRSPLLARLQHLAESWGGKE	174
dHUWE1      IEHLTVLLSSQNMDIVLAVLNLLYMFSKRSNFIP-RLPFEKKELLIVKLFNIAERWGDPN	175
cEEL-1      VDRILALLESTDMDLLAEVLRLLQVMGKRSKFLSTRIPQKEQHALAQRLTAIAQCWGGKL	176
            :::: .** * :*:::  **.** ::.***:::  *:  .::  *  :*  :*: **.
```

```
hHUWE1      NGFGLAECCRDLHMMKYPPSATTLHFEFYADPGAEVKIEKRTTSNTLHYIHIEQLDKISE	234
mHUWE1      NGFGLAECCRDLQMLKYPPSATTLHFEFYADPGAEVKIEKRTTSNTLHYIHIEQLDKISE	234
zHUWE1      NGFGLAECCRDLPMSKYPPSATTLHFEFYAEPSSEVKVEKKSSSNTLHYIHIEQLDKISE	234
dHUWE1      YGLSLKDCCIGEPKLEFLYQ---LCIDYVDEHGHAAQLEIPDMM---------DLCHTAS	223
cEEL-1      RTVKMAECLKREPKLPMLFP-----FTYTDAKQRTIIVEQPKKDESVGELITRTVAQI--	229
              . : :*                 : :         :*               : :
```

```
hHUWE1      SPSEIMESLTKMYSIP-KDKQMLLFTHIRLAHGFSNHRKRLQAVQARLHAISILVYSNAL	293
mHUWE1      SPSEIMESLTKMYSIP-KDKQMLLFTHIRLAHGFSNHRKRLQAVQARLHAISILVYSNAL	293
zHUWE1      SPSEIMESLTAMYNIP-KDKQILLFTHIRLAHGFSNHKRRLQAVQARLHAISILVYSNAL	293
dHUWE1      A-PEVIKTIAGQISKPSEAIKMRIAHRVRLISGFNNYKLRLQFVQARLQAVSILIYSNAL	282
cEEL-1      SPPVSSDSVPS--QQFSKEDLYCLLSRVRMLVTFEDWHHRFKCLIVRLLSVSTLVYCRLG	287
            :     .::    .   :     :  ::*:   *.: : *:: : .** ::* *:*..
```

```
hHUWE1      ---QESANSILYNGLIEELVDVLQITDKQ--------LMEIKAASLRTLTSIVHLERTPK	342
mHUWE1      ---QESANSILYNGLIEELVDVLQITDKQ--------LMEIKAASLRTLTSIVHLERTPK	342
zHUWE1      ---QESANSILYNGLIEELVDVLQITDKQ--------LVDIKAASLRTLTSIVHLERTPK	342
dHUWE1      ---QDNTDKVLYAGFGEELCELIDKEDVH--------LVEIRAAVLRTLTSMLHFDRNPN	331
cEEL-1      GTDETTLSSLLYSGFIEEIVEMLKSDREADTQQDHNLHDAIQTEALSTLCSIVTYEKEPK	347
               : . ..:** *: **: :::.                *::  * ** *::  :: *:
```

```
hHUWE1      L--------SSIIDCTGTASYHGFLPVLVRNCIQAMID-PS--MDPYPHQFATALFSFLY	391
mHUWE1      L--------SSIIDCTGTASYHGFLPVLVRNCIQAMID-PS--MDPYPHQFATALFSFLY	391
zHUWE1      L--------SNIIDCTGTASYHGFLPVLVRNCIQAMID-PL--MEPYPHQFATALFSFLY	391
dHUWE1      VPSRAGSRLMKIVHYTGAERQGGTLPLLVRDCIDNLTTHGL--TERYPLILATSLFSLLY	389
cEEL-1      I--------TQILEALSAGSYHGFLSVMTRQIVDELKSNNLGKTGKPSVSLATALFSFIY	399
            :         .*:.  .:    * * ::.*: :: :              :**:***::*
```

```
hHUWE1      HLASYDAGGEALVSCGMMEALLKVIKFLGDEQDQITFVTRAVRVVDLITNLDMAAFQSHS	451
mHUWE1      HLASYDAGGEALVSCGMMEALLKVIKFLGDEQDQITFVTRAVRVVDLITNLDMAAFQSHS	451
zHUWE1      HLASYDAGGEALVSCGMMEALLKVIKFLGDEQDQITFVTRAVRVVDLITNLDMAAFQSHG	451
dHUWE1      HLASYEMGGTALVKSGMMQSLLCVISWPGVDLEHITFVTRAVRVIDLITNIDIARFHQNN	449
cEEL-1      HLASIEPGGDTLVGSGLTQTLLSVIGFHELPIECITFGTRCARIIDLFTTIDVTSFKANN	459
            **** : ** :** .*: ::** ** :     : *** **..*::**:*.:*:: *: :.
```

```
hHUWE1      GLSIFIYRLEHEVDLCRKECPFVIKPKIQRPNTTQEGEEMETDM----------------	495
mHUWE1      GLSIFIYRLEHEVDLCRKECPFVIKPKIQRPSTTQEGEEMETDM----------------	495
zHUWE1      GLSIFICRLEHEVDLSRKECPFVIKPKIQRPSTATETEDMDTDMDMSEVAMESSPG----	507
dHUWE1      GLNVFIDRLEKEIKSCCKALADIGIT--LKESTLRDDHKLDTSLDHVDEDVEDGSGENAS	507
cEEL-1      GMEICVNRVVHEINECRKEQPFMIDISYDMPFEEEPEPRDDQE---AHEEPEDESDERNP	516
            *:.: : *: :*:. . *    :                 : .
```

```
hHUWE1      ------------------------------------------------------------	495
mHUWE1      ------------------------------------------------------------	495
zHUWE1      ------------------------------------------------------PSTSSG	513
dHUWE1      VTSGNASPRRDNSSDGATGAGSIVYRAYHEHIGGAGSGAGLLDIGGARSSLSGGSSGTSG	567
cEEL-1      VDQEAPVPIEVD------------------------------------------------	528
```

```
hHUWE1      -------------------------------------------------DGVQCIPQRAA	506
mHUWE1      -------------------------------------------------DGVQCIPQRAA	506
zHUWE1      SRAETDSR-------------------------------AQSNASSTPRAGVQCIPQRAA	542
dHUWE1      MGAGSINRDGSAARPPA--IMYPTNSYYARPLAERKAELSSQLPSSLAPKKPSCVTQRAA	625
cEEL-1      -----------APLPPASPIAAPAPVPVASASTSSDSKIKGGPWEEVQMNGATCHQQRSG	577
                                                                 *  **:.
```

```
hHUWE1      LLKSMLNFLKKAIQDPAFSDGIRHVMDGSLPTSLKHIISNAEYYGPSLFLLATEVVTVFV	566
mHUWE1      LLKSMLNFLKKAIQDPAFSDGIRHVMDGSLPTSLKHIISNAEYYGPSLFLLATEVVTVFV	566
zHUWE1      LLKSMLNFLKKAIQDPAFSDGIRHVMDGSLPTSLKHIISNAEYYGPSLFLLATEVVTVFV	602
dHUWE1      LLKSMLNFLKKSIQDHAHFSNMRNIMETSLTQSLRHIIANAEYYGPSLFLLATDVVTVYV	685
cEEL-1      LIKGLLTFIKRAIGDVQFQDIMKHLMEGDLPEALMHILSNAEYYSPSLFHQSAHLITNFV	637
            *:*.:*.*:*::* *  . . ::::*: .*  :* **::*****.****  ::.::* :*
```

```
hHUWE1      FQEPSLLSSLQDNGLTDVMLHALLIKDVPATREVLGSLPNVFSALCLNARGLQSFVQCQP	626
mHUWE1      FQEPSLLSSLQDNGLTDVMLHALLIKDVPATREVLGSLPNVFSALCLNARGLQSFVQCQP	626
zHUWE1      FQEPSLLSSLQDNGLTDVMLHALLIKDVPATREVLGSLPNVFSALCLNARGLHSFVQCQP	662
dHUWE1      FNEPSLLSSLQDLGITSVMLKALLQKDVPATREVLGSLPNVFSALCLNERGLFEFLSYDP	745
cEEL-1      YQFPEELSSIQRRHVPYVIFQSLLRKELPNSKDVITTLGNVFTAMCLNERGLRQFKSYDP	697
            :: *. ***:*   :  *::::** *::* :::*: :* ***:*:*** *** .* . :*
```

```
hHUWE1      FERLFKVLLSPDYLPAMRRRRSSDPLGDTASNLGSAVDELMRHQPTLKTDATTAIIKLLE	686
mHUWE1      FERLFKVLLSPDYLPAMRRRRSSDPLGDTASNLGSAVDELMRHQPTLKTDATTAIIKLLE	686
zHUWE1      FERLFKVLLSPDYLPAMRRRRSSDPLGDTASNLGSAVDELMRHQPTLKTDATTAIIKLLE	722
dHUWE1      FDKVLKVLLSPDYLVAMRRRRSSDPLGDTATNLGNAMDDLIKHHPYLRADATEAIVRLLN	805
cEEL-1      FNQIFRIVLSVKFLVTLRKKRSAEVF-ESAQAIGGALDDLMRHYPDLKHDMLKSIIVILD	756
            *:::::::** .:* ::*::**:: : ::*  :*.*:*:*::* * *: *   :*: :*:
```

```
hHUWE1      EICNLGRDPKYICQK-PSIQKADGTAT------------APPPRS---------------	718
mHUWE1      EICNLGRDPKYICQK-PSIQKADGTAT------------APPPRS---------------	718
zHUWE1      EICNLGRSPEYICQK-PSIQKADGTVT------------VPPARS---------------	754
dHUWE1      ELVRLGSDPSFICWR-ANKESSGSGSGSGSHGGGHHVASTPSPMVMVAGTGS--------	856
cEEL-1      LLDEIGRNPPAGVELVPTLTKTMARS----------VVFSPNNSSLLPREAREARELRIQ	806
             : .:* .*        .  .: .                *
```

```
hHUWE1      --------------------NHAA-EEAS-SEDEEEEEVQAMQSFNSTQQN---------	747
mHUWE1      --------------------NHAA-EEAS-SEDEEEEEVQAMQSFNSAQQN---------	747
zHUWE1      --------------------NHAA-EEAS-SEDEEEEEAL--HTF-SQQQG---------	780
dHUWE1      --------------ASSVLQGAADTNDSS-GDDDDDDD--EMSSASQQQQQPTTPGQG-G	898
cEEL-1      QLQREIPEQADERVGGDVEEEPMEVEDAEDSDVDEEDNEMSMDEMSG--EAPAPPAAPSP	864
                                     :::. .: :::::           :
```

```
hHUWE1      -------------------------ETEPNQQVVGTEERIPIPLMDYILNVMKFVESILS	782
mHUWE1      -------------------------ETEPNQQVVGTEERIPIPLMDYILNVMKFVESILS	782
zHUWE1      -------------------------EPESNRQVVGTEERIPIPLMDYILNVMKFVESILS	815
dHUWE1      GPSTPRTQQAGGVVGSGAGATGTPAAASQAVKVVTPPEREAIPLIDYILNVMKFIEAIFS	958
cEEL-1      KPTKPF-------------------EYGAGMMCEDANGKKILPIGDYMLLIARVVETMMT	905
                                                  :  :*: **:* : :.:*::::
```

```
hHUWE1      NNTTDDHCQEFVNQKGLLPLVTILGLPNLPIDFPTSAACQAVAGVCKSILTLSHEPK--V	840
mHUWE1      NNTTDDHCQEFVNQKGLLPLVTILGLPNLPIDFPTSAACQAVAGVCKSILTLSHEPK--V	840
zHUWE1      NNTTDDHCQEFVNQKGLLPLVSILGLPNLPIDFPTSAACQAVAGVCKSILTLSHEPK--V	873
dHUWE1      NSPNGDHCREFVLHGGLKPILQLLSLPNLPVDSPVSTTSQAVANVCKAILSQAQETK--V	1016
cEEL-1      QSPSQKITEEFIDECVMQKIMKLCHLPCITNEATHATYIGSIANIIKHIVKAGYQSQRNG	965
            :. . .  .**: .  :  :: :  ** :  :   ::   ::*.: * *:. . : :
```

```
hHUWE1      LQEGLLQLDSILSSLEPLH-RPIESPGGSVLLRELACAGNVADATLSAQATPLLHA----	895
mHUWE1      LQEGLLQLDLILSSLEPLH-RPIESPGGSVLLRELACAGNVADATLSAQATPLLHA----	895
zHUWE1      LQEGLCQLDSILSALEPLH-RPIEVPGGSVLLRELANAGHVTDATLSARATPLLHA----	928
dHUWE1      LDVALKQLADIVAQLKPLI-KHFTFPGGSVLLAELVCCQRLEDGFANAEYTPILHN----	1071
cEEL-1      PDVGPKLMSAIVGEFIKTINPLFEVNNGDFSAMNQAE---------SRKATLLMHLDEKL	1016
             : .   :  *:. :       :   .*..   : .          . . * ::*
```

ARLD2 start

**G930D**

```
hHUWE1      ----LTAAHAYIMMFVHT------CRVGQSEIRSISVNQWGSQ--LGLSVLSKLSQLYCS	943
mHUWE1      ----LTAAHAYIMMFVHT------CRVGQSEIRSISVNQWGSQ--LGLSVLSKLSQLYCS	943
zHUWE1      ----LTAAHAYILMFVHT------CRVGQSEIRAISVNQWGSQ--LGLSVLNKLSQLYCS	976
dHUWE1      ----MSFVHGYVVMLVHL------CRNASNDMRTILLKRWGVNRETGVQLLQQLVQLYIS	1121
cEEL-1      VEKTLVELNSIIPTLLNLNKSPMSSTYNPQDHRTRIYETWKMP--EGMKLYGMLRKLSRM	1074
                :   :. :  :::       .    .: *:   : *      *:.:   * :*
```

**S990***

```
hHUWE1      LVWESTVLLSLCTPNSLPSG-CEFGQADMQKLVPKDEKAGTTQG-GKRSDGEQDGAAGSM	1001
mHUWE1      LVWESTVLLSLCTPNSLPSG-CEFGQADMQKLVPKDEKAGTTQG-GKRSDGEQDGTAGSM	1001
zHUWE1      LVWESTVLLSLCTPNSLPPG-CEFGQADMQKLVPKEDKPSSSTAATGRRTAETESLSAGV	1035
dHUWE1      LVWESTILLNLCSDEPTQHPDLIWDEIA----------AQM-SAA-----------AGTA	1159
cEEL-1      LCWEYELVQTLKPTIRTAATQT---EGEMLSDQPADTHLD----------------VEVM	1115
            * **  :: .*              :
```

```
hHUWE1      DASTQGLLEGIGLDGDTLAPMETDEPTASDSKGKSKITPAMAA----RIKQIKPLLSASS	1057
mHUWE1      DASAQGLLEGIELDGDTLAPMETDEPSSSDSKGKSKITPAMAA----RIKQIKPLLSASS	1057
zHUWE1      DPSAQGLLEGMGLDGDSLASMETDEPTATDPKTKSKLTPAMAT----RIKQIKPLLSASS	1091
dHUWE1      DPLTE---------------AELSRKLERAAEFRTKYTP--EE----QFRYIKSLLAASS	1198
cEEL-1      PPWDE----------DSVDE-QQKRDEAKEPRWKAAGVSQEEHDFWIKNKPLQDIVSKSH	1164
                :                : ..      . ::  .         : : :: ::: *
```

```
hHUWE1      RLGRALAELFGLLVKLCVGSPVRQRRSHH--AASTTTAPTPAARSTASALTKLLTKGLSW	1115
mHUWE1      RLGRALAELFGLLVKLCVGSPVRQRRSHH--AASTTTAPTPAARSTASALTKLLTKGLSW	1115
zHUWE1      RLGRALAELFGLLVKLCVGSPVRQRRSHH--TTSTGTAPTPAARATASSLTKLLTKGLSW	1149
dHUWE1      RLGRALAELLGTLVKLSVGSPQTRQRRINDLISNITKVPTPEAREIARILSSILVNGFSH	1258
cEEL-1      AMVKDLLNSMGKT--INVNNPRRPR------IRDTPPLPLAASHCI-SMIFSSIYKDLKW	1215
             : : * : :*    : *..*   :        .    *   ::     : . : :.:.
```

```
hHUWE1      QPPPYTPTP-RFRLTFFICSVGFTSPMLFDERKYPYHLMLQKFLCSGGHNALFETFNWAL	1174
mHUWE1      QPPPYTPTP-RFRLTFFICSVGFTSPMLFDERKYPYHLMLQKFLCSGGHNALFETFNWAL	1174
zHUWE1      QPPPYTPTP-RFRLTFFICSVGFTSPMLFDERKYPYHLMLQKFFCSGGHDALFETFNWAL	1208
dHUWE1      ESILPKPVP-KLKLTFLICSVGFTGPMLFDDKRSAFHLMISQFCAENGLKAFFEMFYWAL	1317
cEEL-1      EPPVQNAATSPMAYGRYIELLTQLNHSLFENGRCSNPALPQNFYTSGCHKAFFELFSDKI	1275
            :    . .   :     *  :   .  **:: :     : .:*  ..  .*:** *   :
```

```
hHUWE1      SMGGKVP--------------VSEGLEHSDLPDGTGEFLDAWLMLVEKMVNPTTVLESPH	1220
mHUWE1      SMGGKVP--------------VSEGLEHSDLPDGTGEFLDAWLMLVEKMVNPTTVLESPH	1220
zHUWE1      SMGGKVP--------------VSEGLEHSELPDGTGEFLDAWLMLVEKMVNPSTVLDSPH	1254
dHUWE1      SLDNVSQKFEFDQWASMEDLMQSHEDDKRDCPEGTGEFLDAWLQLLEKMVNPRAMLDSPY	1377
cEEL-1      I-----P------------------FLGDNMPEGVEQTMEEWCRLAVKLTDRNTIVSSDN	1312
                                         : *:*. : :: *  *  *:.:  :::.*
```

**A1238T**

```
hHUWE1      SLPAKLPGGVQNFPQFSALRFLVVTQKAAFTCIKNL---------WNRKPLKVYGGRMAE	1271
mHUWE1      SLPAKLPGGVQSFPQFSALRFLVVTQKAAFTCIKNL---------WNRKPLKVYGGRMAE	1271
zHUWE1      SLPAKVPGATFTAPQFSALRFLIVTQKAAFSCIRNL---------WNRKPLKVYGGRMAE	1305
dHUWE1      TMSPRLNYMPDSV-PFEPVFYLIHIHQLAMQALRRI---------WARRPIPNYGPSMFE	1427
cEEL-1      WQRRD-----RQSADFDTNKYLKLVCRDMFNAYKQFFEKMAQMPDWELKSLKKVCENAFS	1367
                           *.   :*    :  : . :.:         *  : :        .
```

```
hHUWE1      SMLAILCHILRGEPVIRERLSKEKEGSRGE-EDT--------------------------	1304
mHUWE1      SMLAILCHILRGEPVIRERLSKEKEGSRGE-EEA--------------------------	1304
zHUWE1      SMLAILCHILRGEPIIQERLSKEREGTARP-EEESSSASTAPASGPSGTAGVVGEAVAAG	1364
dHUWE1      TMILILKHLIKSHQTLLDRYHTRMKEIKFE-N----------------------------	1458
cEEL-1      VFKEVAKNLVE-----------ETEATTAATEAVNAAAAPAPADAPAAEWGPGALRIPPV	1416
             :  :  :::.           . :      :
```

**M1328V**

```
hHUWE1      ------------GQEEGGSRREPQVNQQQLQQLMDMGFTREHAMEALLNTSTMEQATEYL	1352
mHUWE1      ------------GQEEGGSRREPQVNQQQLQQLMDMGFTREHAMEALLNTSTMEQATEYL	1352
zHUWE1      ANGNTAGGSTEEASNSTTARREPQVNQAQLTQLMDMGFSREHAMEALLNTSTMEQATEYL	1424
dHUWE1      ---------------LYIRSTDDGLNADHIKTLTDMGFMHYHVIEALRTNASLEEATDYL	1503
cEEL-1      AVE------PAPAPVAVNPAAAADPHEDTIVMLMDLGFSRDIVLYALESTRNADEAANYL	1470
                                     :   :  * *:** :  .: ** .. . ::*::**
```

UBA start

```
hHUWE1      LTHPPPIMGGVVRDLSMSEE---DQMMRAI------AMSLGQDIPMDQRAE---------	1394
mHUWE1      LTHPPPIIGGVVRDLSMSEE---DQMMRAI------AMSLGQDIPMDQRAE---------	1394
zHUWE1      LTHPPPLLSAAVREFTMSEE---DQMMRAI------AMSLGQEVSMEQRSD---------	1466
dHUWE1      LNNPEASSLSTTGGQSSSSAPPPPPPPSAS------TMDIDVDVPADGEST---------	1548
cEEL-1      LAHGNEIPLQDAANPF------RDDLLNQVLREAGAEMIAGANTPNADEIELQLNAQFAE	1524
            * :        .                         *  . :     .
```

UIM start

```
hHUWE1      -SPEEVACRKEEEERKARE---------------------------KQEEEEAKCLEKFQ	1426
mHUWE1      -SPEEVACRKEEEERKARE---------------------------KQEEEEAKCLEKFQ	1426
zHUWE1      -SPEEAARRREEEERRARE---------------------------RVEEEEARCLERFL	1498
dHUWE1      -QSK--------------S------------------------------STSPNSSYDYK	1563
cEEL-1      HQGYELVRIAQRVLRDAGAEQIGLQIMEAYPDESVDVAAEQLINDVMDRVRNPRSSYEEN	1584
             .                                                 . ..
```

```
hHUWE1      DA----------DPLEQDELHTFTDTMLPGCFHLLDELPDTVYRVCDLIMTAIKRNGADY	1476
mHUWE1      DA----------DPLEQDELHTFTDTMLPGCFHLLDELPDTVYRVCDLIMTAIKRNGADY	1476
zHUWE1      EA----------EPLDTTELHAFTDSMLPGCFHLLDELPDTVYRLCDLLMTAIKRNGPEY	1548
dHUWE1      HL----------KLMPSLIFDRFCDEAISRAFEFMEAIPDTVNSAADLIAVLYRRHNHLN	1613
cEEL-1      IAQPLIPPLSQLKIEQDVSLNSACKQLFPLVKRLLLVSNDTIHPCAELIVSIFPAM----	1640
                        .      :.   .  :    .::    **:   .:*:
```

```
hHUWE1      RDMILKQVVNQVWEAADVLIKAALPLTT--SDTKTVSEWIS----QMAT----LPQASNL	1526
mHUWE1      RDMILKQVVNQVWEAADVLIKAALPLTT--SDTKTVSEWIS----QMAT----LPQASNL	1526
zHUWE1      RDLILRQVVNQVWEAADVLIKAAVPLTT--SDTKTVSEWTR----QMAT----LPQASNL	1598
dHUWE1      KQVFVTNFVRNIIQWVDFTLQLFEPAKTTGSKASRLEECLT----GMTA----TRLFVRL	1665
cEEL-1      -----------TEEWRKEHLIA----EICGEDLKNMAKSLVEQEVDENGRPKQHDVARTM	1685
                         :  .  :          .. . : :                     :
```

```
hHUWE1      ATRILLLTLLFEELKLPCAWVVESSGILNVLIKLLEVVQPCL-----QAAKEQKEVQTPK	1581
mHUWE1      ATRILLLTLLFEELKLPCAWVVESSGILNVLIKLLEVVQPCL-----QAAKEQKEVQTPK	1581
zHUWE1      ATRILLLTLLFEELKLSCARVVESSGVLTVLIKLLEVVQPCL-----QAAKEQKDIQTPK	1653
dHUWE1      KTSTLLLEENYSDLHRPLVEAITAQDAMVSLVKLLDCMSQWMLEQSTETNTGQQRATVPR	1725
cEEL-1      TNRLHFAVLIFDKISEEYVQWIDANGMTEVMLTSLEYLVERF----KS------VDYYQS	1735
             .   :    :..:    .  : :..    ::. *: :   :     .
```

```
hHUWE1      WITPVLLLIDFYEKTAISSKRRAQMTKYLQSNSNNWRW-------------FDDRSGRWC	1628
mHUWE1      WITPVLLLIDFYEKTAISSKRRAQMTKYLQSNSNNWRW-------------FDDRSGRWC	1628
zHUWE1      WITPVLLLIDFYEKMAVSSKRRAQMNKYLQPNGNNWRW-------------FDDRSGRWC	1700
dHUWE1      WVQNLVDLIDAIDSISHILQRKANMR---AVSSDVWRW-------------YDASTGKWN	1769
cEEL-1      LITRIICWNDFYAKTHRLIVRRAYLKSL--SPSLVWSYQSYEEDALRRRADYSSGEKKWV	1793
             :  ::   *   .      *:* :       .  * :             :.    :*
```

WWE start

```
hHUWE1      SYSASNNSTIDSAWKSGETSVRFTAGRRR-----YTVQFTTMVQVNEETGNRRPVMLTLL	1683
mHUWE1      SYSASNNSTIDSAWKSGETSVRFTAGRRR-----YTVQFTTMVQVNEETGNRRPVMLTLL	1683
zHUWE1      SYSASNNSTIDSAWRAGETSVRFTAGRRR-----YTVQFNTMVQVNEETGNRRPVMLTVQ	1755
dHUWE1      AYSEANNELIRNAYAAGERWLYINIGRQR-----CTVSLNCMTQVSEASGTHRPVCPALK	1824
cEEL-1      PYDAASQKTLNDAFFAGVRGVKCTLKRGVRPNKKVDVDFASM------------------	1835
             *. :.:. : .*: :*   :  .  *         *.:  *
```

```
hHUWE1      RVPRLNKNS-------------------KNSNGQELEKTLEESKE----MD---IKR---	1714
mHUWE1      RVPRLSKNS-------------------KSSNGQELEKTLEESKE----TD---IKH---	1714
zHUWE1      RVPRIPKAT-------------------KSGSVTDTEREESEQRA----KT---E-E---	1785
dHUWE1      LSEAITSLNNPVALHKVEHILNRVVRTAPDGSGTVHSDELISLRQLVNFTDGQQPQQSEN	1884
cEEL-1      ----------------------------KQGDGTSRENIK--------------------	1847
                                         ...    .


hHUWE1      -----KENKGNDTPLALESTN----------------TEKETSLEETKIGEI-LI-----	1747
mHUWE1      -----KENKGNDIPLALESTN----------------TEKEASLDETKIGEI-LI-----	1747
zHUWE1      -----TQTDTDSSSVAVEMAPPKEES----ELKDSTPAAPSVLQDPSGSSGI-VV-----	1830
dHUWE1      SANPSEEATSEDGPAAANSGSPPATMTTRSKTRQKSAAAAAAAKNKSQSGGSPPIPKRTQ	1944
cEEL-1      ----------AELPPTVEVSI-------------------------------PDLMDAEA	1866
                       .   : :                                    :


hHUWE1      -QGLTEDMVTVLIRACVSMLGVPV---------DPDTLHATLRLCLRLTRDHKYAMMFAE	1797
mHUWE1      -QGLTEDMVTVLIRACVSMLGVPV---------DPDTLHATLRLCLRLTRDHKYAMMFAE	1797
zHUWE1      -QGLTEDMITILIRACVSMISVPV---------DPDTLHATLRLCLRLTRTHHYAMMFAE	1880
dHUWE1      KIILNEEQVGLSKFDCGRIIGSIVDLLQPSHQMLNETKLSLLRLCARLTRNYDNAQVFIR	2004
cEEL-1      KLSWSEDQND-------RFLDLSTQ-ILRTGALDPKCSHSMLSFIARLTRSHRNAVRFME	1918
                .*:           ::.  .           .   : * :  **** :  *  * .
```

ARLD3 start

```
hHUWE1      LKSTRMILNLTQSSGFNGFTPLVTLLLRHIIEDPCTLRHTMEKVVRSAATSGAGSTTSGV	1857
mHUWE1      LKSTRMILNLTQSSGFNGFTPLVTLLLRHIIEDPCTLRHTMEKVVRSAATSGAGSTTSGV	1857
zHUWE1      LKSTRMILNLTQSSGFNGFTPLVTLLFRHIIEDPATLRHTMEKVVRSAVTSGAGSTTSGV	1940
dHUWE1      RGGVRMLLHLQQACSFMGFPTYANIILRHVLEAPPVLQEAMERVLGMRA--------AGI	2056
cEEL-1      KDGVEAILRLRARCAI-TYPFLVSIIIRNCIDDDALLGHIYEKTIRGYIAVPTQPPASMS	1977
              ... :*.*   ..:  :   ..:::*: ::    * .  *:.:            :
```

HWA start

```
hHUWE1      VSGSLGSREINYILRVLGPAACRNPDIFTEVANCCIRIALPAPRGSGTASDDEFENLRIK	1917
mHUWE1      VSGSLGSREINYILRVLGPAACRNPDIFTEVANCCIRIALPAPRGSGTASDDEFENLRIK	1917
zHUWE1      VSGSLGSREINYILRVLGPAACRNPECFTETAKSCIRIALPAPRGAGTASDDEFENFRIK	2000
dHUWE1      --VPVGHRDLIYVLTQVSSAVSRNPQIFVAAAKEMLKGEYLINTNSTTLADDARVMVKSK	2114
cEEL-1      EYAAGKSKDFADTLNFMAPLSTRNPLVFTEAMNKLARMNGSLI----------LPMIKEK	2027
                   :::   *  :.    ***  *. . :   :                   .: *
```

```
hHUWE1      -G--PNAVQLVK-TTPLK--PSPLPVIPD-------------TIKEVIYDMLNALAAYHA	1958
mHUWE1      -G--PNAVQLVK-TTPLK--PSSLPVIPD-------------TIKEVIYDMLNALAAYHA	1958
zHUWE1      -G--PNAVQLVK-TTPLK--LSPLPSIPE-------------TIKEVIYDMLNALAAYHA	2041
dHUWE1      FGQQPTA-AAGG-ATPHNQTPTPPPAAKDELKDDPQVQSSVAVLKELLHGLVQPCWHYGG	2172
cEEL-1      KPTLSVSSTAGGASTSSSSAPPPPPPLTSSSSHT-VVEN-NSRAEKIVSMMLSE------	2079
                  :      :*  .      *   .               ::::  ::.
```

```
hHUWE1      PEEADKSDPKPGV--MTQEVGQLLQDMGDDVYQQYRSLTRQSSDFDTQSGFSINSQVFAA	2016
mHUWE1      PEEADKSDPKPGG--TTQEVGQLLQDMGDDVYQQYRSLTRQSSDFDTQSGFSLNSQVFAA	2016
zHUWE1      PEEAERGEERANAVPGNQDLCQILQDVGDDVYQQYR-LTRQGSDFDSQSAFPINTQVFAA	2100
dHUWE1      TGSLDRGQGAA----------ELLQPD-------QPPGTAQGCHDDEETPAPVSSKK---	2212
cEEL-1      ----------------------V-------------------------------------	2080
                                  :
```

```
hHUWE1      DGASTETSASGTSQGEA-STPEESRDGKKDKEGDRAS---EEGKQKGKGSKPLMPTSTIL	2072
mHUWE1      DGAPAETSTTGTSQGEGASTPEETREGKKDKEGDRTS---EEGKQKSKGSKPLMPTSTIL	2073
zHUWE1      DGAVAESSQSGTPQGEA-STPEEMREEKKEQEGDKGASSEESRAAKAKASKPLMPTSTIL	2159
dHUWE1      ----------QSSSGGGAAAAGQTGQ--ATEKFEPCCCTWHIPTSGHTHAKPTISRATLL	2260
cEEL-1      ----------------------------------------LNGEFPTVGQTRMLSQEKIL	2100
                                                              .  :   .:*
```

```
hHUWE1      RLLAELVRSYVGIAT-LIANYSYTVGQSELIKE-DCSVLAFVLDHLLPHTQNAEDKDTPA	2130
mHUWE1      RLLAELVRSYVGIAT-LIANYSYTVGQSELIKE-DCSVLAFVLDHLLPHTQNAEDKDTPA	2131
zHUWE1      RLLAELVRSYVGIAT-LITSYNYTAGQSELIKE-DCSVLAFVLDHLLPHTQNSEDKDTPA	2217
dHUWE1      KLLADSTRAYQSLVPRVLLSHVYTPADSPLISAPQQTFLGVLLDRFLPITQRHHVPEVST	2320
cEEL-1      QILAEIVKSYPSLAIIIA--ETQAEGRSALHS---------LIDTYIIAPI--EKIETTN	2147
            ::**: .::* .:.  :      : . * * .         ::*  :      .  :.
```

**R2162W**

```
hHUWE1      LARLFLASLAAAGSGTDAQVALVNEVKAAL--------------GRALAMAES-------	2169
mHUWE1      LARLFLASLAAAGSGTDAQVALVNEVKAAL--------------GRALAMAES-------	2170
zHUWE1      LARLFLASLAAAGTGTDAQAALVNEVKAAL--------------SRALGMAEG-------	2256
dHUWE1      MTRVLICSLSDCYQQPGVQVHVAEELHAAV--------------ARALAQPDS-------	2359
cEEL-1      ALKTLIAVISASQNSLKAQELLVLDVKNALASYSEKASELRVDLAKLQLDKESEEREEAT	2207
              : ::. :: .     .*  :. ::: *:              .:     :.
```

```
hHUWE1      -------TEKHA------RLQAVMCIISTIMESCPSTSSFYSSATAKTQHNGMNNIIRLF	2216
mHUWE1      -------TEKHA------RLQAVMCIISTIMESCPSTSSFYSSATAKTQHNGMNNIIRLF	2217
zHUWE1      -------TEKHA------RLQAVMCIISTIMESCPSTSSFYSTATAKTQHNGMNNIIRLF	2303
dHUWE1      -------AEKHT------RLQVLMSLFPNLIESPM----LY-----DKVHMHRNNMYRVL	2397
cEEL-1      TSLKKQETEVLSKISELCSIIIIMCQSCPAHHH----HHHSSTDRNNRERSSQNAIMKMF	2263
                   :*  :       :  :*.      .              .  :   * : :::
```

```
hHUWE1      LKKGLVNDLARVPHSLDLSSPNMANTVNAALKPLETLSRIVNQP--------SSLFGSKS	2268
mHUWE1      LKKGLVNDLARVPHSLDLSSPNMANTVNAALKPLETLSRIVNQP--------SSLFGSKS	2269
zHUWE1      LKKGLVNDLARVPHSLDLSSPNMANTVNAALKPLETLSRIVNQP--------SSLFGGKG	2355
dHUWE1      LRQGVMTYLTKVAQYKDLSNHNTLSTLAAILRPMEILLRFSVSTTTL--GSKRILFGSGG	2455
cEEL-1      HKKRMCADLVKTIHCLQLSTKVSLDTVNQILKTLDTLLEGSTTTTSATITGPRSLMDIVA	2323
             :: :   *.:. :  :**.    .*:   *: :: * .               *:.  .
```

```
hHUWE1      ASSKNKSEQ--DAQGASQDS-SSNQQD-------------------------PGEPGEAE	2300
mHUWE1      ASSKNKSEQ--DAQGASQDS-SSHQQD-------------------------PGEPGEAE	2301
zHUWE1      GSSKNKTEH--DTVGTVRDS-NSNTQD-------------------------QGEVGEAE	2387
dHUWE1      PGGPNASGNGNNTAGGSSGNGPGSGNTYGTIINRRLYPRLAARTGFGSAMERSNAMGGEH	2515
cEEL-1      ---------------GRREQ--RREAAAGGIMNER-------------------------	2341
                               .


hHUWE1      VQ---------EEDHD-----------VTQTE-VADGDIMDGEAETDSVVIAGQ------	2333
mHUWE1      VQ---------EEDHD-----------VTQTE-VADGDIMDGEAETDSVVIAGQ------	2334
zHUWE1      PV---------ENR--------------DRGQ-ATDSDLMDGEAEGDTVVIAGQ------	2417
dHUWE1      VLRDIIRNVLSDRRHAFEFIFNSDDMAVDSEDAAAPGSGVGGGGSGAGSVGGGVSGSVSG	2575
cEEL-1      ---------------EIDTIFQRDGLAFDGEMENLLRRLQGDHWRGN-SVQRGA------	2379
                                                    ..       *  *
```

**L2356F**

```
hHUWE1      ----------------------PEVL------------SSQEMQVENELEDLIDELLERD	2359
mHUWE1      ----------------------PEVL------------SSQEMQVENELEDLIDELLERD	2360
zHUWE1      ----------------------PEVL------------STQAMQVENELVDLIDELLERD	2443
dHUWE1      NSSAAVIDVVEERSNGGDTNDPPANVSSASSGAGQSSTSGGPVRPVVNFDQLWDDFLQSE	2635
cEEL-1      -------GQVAEEEEGSET---------------------------------YE---ERE	2396
                                                              :   : :


hHUWE1      GG---------S--GNSTIIVSRSGEDES---------------Q---------------	2378
mHUWE1      GG---------S--GNSTIIVSRSGEDES---------------Q---------------	2379
zHUWE1      AQ---------T--VNSTIIVGRSGEDES---------------Q---------------	2462
dHUWE1      GLRLASRSGGGAGSGSGSVGASSSGQQNGSNTTASSTNSGNNSGIGNLRLRTNPDVDVGS	2695
cEEL-1      RSDS-------------------------------PSESSEHAG----------------	2409


hHUWE1      -----------EDVLMDEAPSN---------LSQASTLQANREDSMNI------------	2406
mHUWE1      -----------EDVLMDEAPSN---------LSQASTLQANREDSMNI------------	2407
zHUWE1      -----------EDVLMDEAPSN---------MSQASTLQANREDSMNI------------	2490
dHUWE1      GNGSGSGSGSNAGVVNDNGISGGGGSDGASTSSDTGAPGGARDLNASLLGD--------V	2747
cEEL-1      -----------DEEVRDDAVETGDGEVDMADAQDAPLAAPDAPPGINLVDEIMQQVQRDV	2458
                          : *:. .           .::         . .:


hHUWE1      ------LDPEDEEEHTQEE----------DSSGSNEDEDDSQDE---EEE----------	2437
mHUWE1      ------LDPEDEEEHTQEE----------DSSGSNEDEDDSQDE---EEE----------	2438
zHUWE1      ------LEPEDE-EHTQEE----------DSSGSNEDEDSQDEE---EEE----------	2520
dHUWE1      STSESDSDPSTENDERNEED------EDEDDDAPDEDVDEHSET-DVDEERSRQFIEVFD	2800
cEEL-1      EDDEEDED-GDQNEHEAEEDDHDREDEDDEDDDDDEDEEEEAEDDDQDEDDVRHV----E	2513
                   :   : :.  **          :..  :** :.  :    :*:


hHUWE1      ---------E---EEDEEDDQEDDEGEEGDEDDDDDGSEMELDEDYPDM-N---------	2475
mHUWE1      ---------E---EEDEEDDQEDDEGEEGDEDDDDDGSEMELDEDYPDM-N---------	2476
zHUWE1      -------------EEE---EEEDQDDEEGDEDDDDEGSEMELDEDFPDI-N---------	2554
dHUWE1      HIYEPESSETASNDADVDNDDDDDEDDVDDDDDDDEDNDDDVREEVQSVMDAEIENRIDI	2860
cEEL-1      --QNPEPLARRLFEED-DDDEEDDDGDEDGDSMEDDVQRLDLDDDYFDMGGPFDAQRMDD	2570
                         : :   :::*::.: ..:. :*: .  :: ::  .: .


hHUWE1      --------ASP----------LVRFERFDREDDLIIEFDNMFSSATDIPP----------	2507
mHUWE1      --------ASP----------LVRFERFDREDDLIIEFDNMFSSATDIPP----------	2508
zHUWE1      --------AAP----------HIRFERFDRDDDLIIEFDNMFSTSADIPP----------	2586
dHUWE1      ALALVSSNNRPRRSAAVNASGDGSANRTNPDDEVDDE-------DDDVDA----------	2903
cEEL-1      MI-FPPSFGRP----------------------AVTSFADLFRDDFDFLPPYRAERRPLA	2607
                      *                         .         *.


hHUWE1      ---SPGNIPTTHPLM--------------------------VRHADHS------SLTLGS	2532
mHUWE1      ---SPGNIPTTHPLM--------------------------VRHADHS------SLTLGS	2533
zHUWE1      ---SPGNIPSSHPLM--------------------------VRHADHG------SLTLGG	2611
dHUWE1      ---DPDGDPDVDPISGPSAAQAEAYLYERLGSGLMPTPPVGFRVRMNSSIAQPLDIDVNG	2960
cEEL-1      RGSHLGGVISEHPLMTR--------------------PPAENDVS---------------	2632
                 ..    .*:


hHUWE1      GSSTTR-----LTQGIGRSQRTLR-QLTA------------NTGHTIHVHY-----P---	2566
mHUWE1      GSSTTR-----LTQGIGRSQRTLR-QLTA------------NTGHTIHVHY-----P---	2567
zHUWE1      SGTSSR-----LAQGMGRSQRSLR-QLTA------------NTGHTIHVHY-----P---	2645
dHUWE1      GNAGSGSNAGATGSSGGSGQRSSTGGTTASIGAIASAALRPSSGSWMAPDFNFIRISGGD	3020
cEEL-1      ----RRPNT----------------NIRAE-------ILQMHTRSSLHRQNAIRRTT---	2662
                                        *             :   :  .


hHUWE1      ----------GNR-------QPNPPLILQRLLGPSAAA-DILQLSSSLPLQSRGR--ARL	2606
mHUWE1      ----------GNR-------QPNPPLILQRLLGPSAAA-DILQLSSSLPLQSRGR--ARL	2607
zHUWE1      ----------GNR-------QPNPPLILQRLLGPSAAA-DILQLSSSLPLQSRGR--ARL	2685
dHUWE1      SSMGSGPAIGGNANGGDMNVFITPSMSLERAGGAPGSGQTMSELLNEQPTGAGGGGGGGS	3080
cEEL-1      --------------------------TEARELEAISRGIRMGE----LPAG-------RM	2685
                                         *     . .  : :     *


hHUWE1      --LVGN-------DDVHI---IA----RSDDELLDDFFH----DQ----------STATS	2636
mHUWE1      --LVGN-------DDVHI---IA----RSDDELLDDFFH----DQ----------STATS	2637
zHUWE1      --MVGN-------EDVHI---IA----RSDDELLDDFFH----EQ----------SSTGG	2715
dHUWE1      ASGVGGVGANGGTTGQHPNATLCTNGVRRRLLYLDQQYCVCMPTH----------QNPTE	3130
cEEL-1      --LVRGTT-THGDGGVHT-------------SFFDHIFDIRPSNVAFSRSGAYRSYNTGD	2729
               * .        . *                :*. :                  .


hHUWE1      ----QAGTLSSIPTALTRWTEECKVLDAESMHDCVSVVKVSIVNHLE-FLRDEELEERRE	2691
mHUWE1      ----QAGTLSSIPTALTRWTEECKVLDAESMHDCVSVVKVPIVNHLE-FLRDEELEERRE	2692
zHUWE1      ----QAGTLSSIPTALTRWTDECKVLDAESMHDCVAVVKVPILQHLE-SLRDEELEERRE	2770
dHUWE1      ----ETQMETFTQDALHWWLEEATLLDMESQTDACLYAAHFLLPHLIKELKAAHQQRKQE	3186
cEEL-1      RDERRDIRASQVPTCLERLE--SYSLSMEPVS--SRFVTVIVNSHLNKIHAAREAQIKKE	2785
                .    :    .*      .  *. *        .   :  **       . : ::*
```

Tower start

```
hHUWE1      KRRKQLAEEETKITDKGK--EDKENRDQSAQCTASKSND-----STEQN-----------	2733
mHUWE1      KRRKQLAEEETKIIDKGK--EDKENRDQSAQCTVTKTND-----STEQN-----------	2734
zHUWE1      KRRRQLAEEEETAKQNESSAAGEETREQSLQGSGIGTVN-----GAAEN-----------	2814
dHUWE1      EAARQ-----STTPASGSNASATSSSTTSVVGTAAPTATSSIASGLLANAVSAGSGSSSL	3241
cEEL-1      AEIKAKA--------KKDAEAAKKAAEDKKKAAEAPA-----------------------	2814
               :           . .     .    .   :   :
```

```
hHUWE1      -------LSDGTPMPDSYPTTPSST-------------------------DAATSESKET	2761
mHUWE1      -------VSDGTPMPDSYPTTPSST-------------------------DAPTSESKET	2762
zHUWE1      -------AAEGEQAQSGVS---SCL-------------------------DPP-RTSEGF	2838
dHUWE1      VVPPVAASATGGTRRSSIPVLISSTLGPSSASSATSATGNGATSDATTTTGGPTRNFSS-	3300
cEEL-1      --------------------------------------------------PAPSAEQ---	2821


hHUWE1      LGTLQSSQQQPTLPTPPALGE------VPQELQSPAGE----------------------	2793
mHUWE1      LGTLQPSQQQPALPPPPSLGE------IPQELQSPAEE----------------------	2794
zHUWE1      LTAPPSGEVTPTTPAPHEQALVSLETAISQQVHQPITELLLADSN---------------	2883
dHUWE1      VDSNVSSETQTTTRPPRQRGGLRQQSQLPFNIYAEIDLTNEQDSEGDRSTSTTTGTAADG	3360
cEEL-1      SDSPA--SPDAPAPAPEEIGLATPAA---TNTTNTVS-----------------------	2853
              :    .       *   .          :


hHUWE1      ------------------GGSSTQLLMPVEPEELGPTRPSGEAETTQME-----------	2824
mHUWE1      ------------------VANSTQLLMPIELEELGPTRPSGEAETTQME-----------	2825
zHUWE1      ------------------PDSHGALAGAGLPQLSAPDRPNCEAEASQME-----------	2914
dHUWE1      AESQSQHNSSSTAAQVAVPQHQRHPPVPLLPSSGRPSRPHTRLDETELLLLQLTDRQIRN	3420
cEEL-1      ------------------------------------------------------------	2853


hHUWE1      -------------------------------------------------LSPAPTIT---	2832
mHUWE1      -------------------------------------------------LSPAPTIT---	2833
zHUWE1      -------------------------------------------------MSPAPTIGERG	2925
dHUWE1      RLQGSSGHTNSSRTSGSALINARPNRDLPACLQRHDHPAGSIFPRPSANRTPAPTLVSMP	3480
cEEL-1      -------------------------------------------------ESVAPNASTRG	2864
                                                              : **.


hHUWE1      -------------------------SLSPERAEDSDALTA--VSSQLEGSPMDTSSLASC	2865
mHUWE1      -------------------------SLSPERAEDSDALTA--VSSQLEGSPMDTSSLASC	2866
zHUWE1      ----GGALDA--------V---REASLSPDIAESSEPVPV--GVSQLEGSPMDTSSPASG	2968
dHUWE1      TPMSNAALEQLNVTAPDVVRTDRNFALPSSLEVDVDVLPGIVDLPQQEAQPEDDQLLLPP	3540
cEEL-1      ---------------------D-----------DTLTTP---AAAQVEDQASEDQHIEEE	2889
                                             .           * * .  : .


hHUWE1      TLEEAVGDTSAAGSSEQP--------RAGSSTPGDAPPAVAEVQG--------RSDGSGE	2909
mHUWE1      TLEEAVGDTPAAGSSEQP--------TAGSSTPGDAPSVVAEVQG--------RPDVSRE	2910
zHUWE1      TQEEPAASSVPSNHMSQE--------LSGSGDSGL-----TDRQT--------DAETGST	3007
dHUWE1      PPPPPPSDTTPLVHAEDD-LWPGTVVSAGTREESSAPAVATESTEAVNESNQPEPTPESS	3599
cEEL-1      PMEVGEDDGA--AEAPQSSAATAASSVAGTEEIED-----VERQA--VEDH--VPEIQDN	2938
                   .        :          :*:          .:


hHUWE1      SAQPPEDSSPPASSESSSTRDSAVAISGADSRGI------------LEEPLPSTSSEEED	2957
mHUWE1      SNQPPEDSSPPASSESSSTRDSAVAISGADSRGI------------LEEPLPSTSSEEED	2958
zHUWE1      SVSSPGETMP------------RSDSADSQSQAI------------QEEPLPSTSNEEED	3043
dHUWE1      ESTSPNPQAPSAEPTPLEPGAAVDASVGAQTPATTEEAASTAGASGTTATITDMSPEVRA	3659
cEEL-1      -----DDTLPY---------------EGAV-----------EHAPAQAPEENVMPEEFRA	2967
                     *                 .:                           * .
```

UBM start

```
hHUWE1      PLAGISLPEGVDPSFLAALPDDIRREVLQNQLGIRPPTRTAPST---N------------	3002
mHUWE1      PLAGISLPEGVDPSFLAALPDDIRREVLQNQLGIRPPTRSAPSS---N------------	3003
zHUWE1      PLAGISLPEGVDPSFLAALPEDIRREVLQNQLGIRPPSRPPVAS---T-LTT--------	3091
dHUWE1      ALGDLEVPEGVDPSFLAALPSEMREEVIQEHLRMQRIRQRAQQ-----------------	3702
cEEL-1      ILGDIIIPDGVDPAFLAALPEEMRAEVIRDYQRQQRAERASRPVPAAQPVAVNPNAPAGG	3027
             *..: :*:****:******.::* **:::    :   :
```

```
hHUWE1      --------SSAPAVVGNPGVTEVSPEFLAALPPAIQEEVLAQQRAE------QQRRELAQ	3048
mHUWE1      --------SSAPAVVGNPGVTEVSPEFLAALPPAIQEEVLAQQRAE------QQRRELAQ	3049
zHUWE1      --------TASAVLGGGPGVTEVSPEFLAALPPAIQEEVLAQQRAE------QQRRELSQ	3137
dHUWE1      ----------NAIQIAHDSLVEVNPEFLAALPLNIQSEVLMQQRIE------QQRQAA--	3744
cEEL-1      EAAQGEGQAEQPAAAVVPLVEPIDPVFLNALPPELQEEVLAEHERRLREAEEQQRRQN--	3085
                               :  :.* ** ***  :*.*** ::. .      ***:
```

**R3070H/C**

**D3068G**

**R3071P**

```
hHUWE1      NA-SSDTPMDPVTFIQTLPSDLRRSVLEDMEDSVLAVMPPDIAAEAQALRREQEARQRQL	3107
mHUWE1      NA-SSDTPMDPVTFIQTLPSDLRRSVLEDMEDSVLAVMPPDIAAEAQALRREQEARQRQL	3108
zHUWE1      QPTQGDQPLDPVTFIQTLPSELRRSVLEDMEDSVLAVMPPDIAAEAAALRREQEARQRQL	3197
dHUWE1      QTANPEDPVDTAAFFQNLPENLRQAILTDMEESQIASLPPELAAEAQFLRRDWESRNGAR	3804
cEEL-1      APPAPVVEMDGAAVIASLPANERAQVLAEMDDAELAGLPADMQNEARRARAQHVEPNMLR	3145
                    :* .:.: .** : *  :* :*::: :* :* ::  **   * :    :
```

Hotspot 2

```
hHUWE1      MHERLFGHSSTSALSAILRS------PAFTS----RLSGNRGVQYTRLAVQRGGTFQMGG	3157
mHUWE1      MHERLFGHSSTSALSAILRS------PAFTS----RLSGNRGVQYTRLAVQRGGTFQMGG	3158
zHUWE1      MHERLFGHSSSSALSAILRS------PAFTS----RLGGNRGVQYTRLAVQRGGTFQMGG	3247
dHUWE1      MDAH----PPSNALSRFQTTLQSLDDTRWHSSIWYDASGNAQPQHH----QHTTIVHQHP	3856
cEEL-1      YHRLLFRGGVGGAPGAIGTVRA---------RTNARAAANAGP-----------------	3179
             .         .* . :                    ..*


hHUWE1      SSSHNRPSGSNV--DTLLRLRGRLLLDHEALSCLLVLLFVDEPKLNTSRLHRVLRNLCYH	3215
mHUWE1      SSSHNRPSGSNV--DTLLRLRGRLLLDHEALSCLLVLLFVDEPKLNTSRLHRVLRNLCYH	3216
zHUWE1      SANHSRPSSSNV--DSLLRLRGRLLLDHEALSCLLVLLFVDEPKLNTSRLHRVLRNLCYH	3305
dHUWE1      HAHHAPNMGKPL--ALLMMEDENILLDPDSLATLLLFLFVEDQKVNSMRLHRVIRNLCHH	3914
cEEL-1      ------SVGQNAGNAIQAPPDQPHLLDRESILTLCLLYLVDNNRVPHTRLQKVLRSACVN	3233
                    ..              *** :::  * :: :*:: ::   **::*:*. * :
```

ARLD4 start

```
hHUWE1      AQTRHWVIRSLLSILQRSSESELCIETPKLTTSEEKGKK------SSKSCG---------	3260
mHUWE1      AQTRHWVIRSLLSILQRSSESELCIETPKLSTSEERGKK------SSKSCA---------	3261
zHUWE1      SQTRGWVIRSLLSILQRSSESELCLETARLEESRGKRSLGQGSSSGGKGCSSSSQSAIAS	3365
dHUWE1      EPTRDWIISALISIIQKTNEDNANFG--------------A-------------------	3941
cEEL-1      QTTCDFIVWCLLALLDKASEANTDDE----------------------------------	3259
              *  ::: .*:::::::.* :
```

```
hHUWE1      --SSSHENRPLDLLHKMESKSSNQLSWLSVSMDAALGCRTNIFQIQRSGGRKHTEKHAS-	3317
mHUWE1      --SSSHENRPLDLLHKMESKSSNQLSWLSVSMDAALGCRTNIFQIQRSGGRKHTEKHAS-	3318
zHUWE1      SSSSSSSSSSLELLNRVESRSSSQLSWLSVSMDAALGCRTNIFQIQRVSGRKHAERHSAG	3425
dHUWE1      ------------------GQAGGKPEWLKLRVDAAFGYKSNIFLINRLYE----------	3973
cEEL-1      ------------------EILSNVPAWLDSIAVSGVGHNERAIRI---------------	3286
                                 ..   **.    :..* . . : *
```

```
hHUWE1      ----------------------GGSTVHIHPQAAPVVCRHVLDTLIQLAKVFPSHFTQQR	3355
mHUWE1      ----------------------SGSTVHIHPQAAPVVCRHVLDTLIQLAKVFPSHFTQQR	3356
zHUWE1      GSVSAGGLTGGVSGTGMTCTGGGGSTVHIHPQAAPVVCRHVLDTLIQLAKVFPSHFTQQR	3485
dHUWE1      ---------------------GSARSISINPQAAQMIVRNCLDLLFVLAKHYPSSFVPFN	4012
cEEL-1      --------------------SENAQKISIHSMLAIPMCKNILDLLANIARAYPGNFLPLI	3326
                                  .. .: *:   *  : :: ** *  :*: :*. *
```

```
hHUWE1      TKETNCESDRERG-----------------------------------------------	3368
mHUWE1      TKETNCESDRERG-----------------------------------------------	3369
zHUWE1      CKDPLSSSSSELDTRLCASSTPTMS-------ASCRSGGTSQSNS---------------	3523
dHUWE1      RDVKARRI---L-SAVFGMPSPTAGSGGGAGSGSAASGGLTGYDDVPPGGTLDFRSRFCR	4068
cEEL-1      LRHGAKPTD---------------------------------------------------	3335
```

**A3371T**

```
hHUWE1      ------------------------------------------N-KACSPCSSQSSSSGIC	3385
mHUWE1      ------------------------------------------SKQACSPCSSQSSSSGIC	3387
zHUWE1      ---------------SATP--------SSL----------TSIHNSLSACSGTPQSLGIS	3550
dHUWE1      YQVVNRLRSIFDDHPAPKPSVPLDEAPSTSKAAAAKAAATAKVSTAGSQNKHSVPYQTNA	4128
cEEL-1      ----------------------------------------------------TPKQAPSF	3343
```

```
hHUWE1      TDFWDLLVKLDNMNVSRKGKN---SVKSVPVSAGGEGETSPYSLEASPLGQLMNMLSHPV	3442
mHUWE1      TDFWDLLVKLDNMNVSRKGKN---SVKSVPVSSGGEGETSPHSLEASPLGQLMNMLSHPV	3444
zHUWE1      TDFWDLLVKLDNMNVSRKGKA---SMKTVPLGGVGETEGALFSLEASPLGQLMNMLSHPV	3607
dHUWE1      KNFWDVVLNIDQQTQVRLANVAQFPLTSQPAWEWQPNTADFLSFSESPFGQLLEMLPYKV	4188
cEEL-1      AQFWTMVQNTTKVPKS-------------KDWTA----TPEQQLEECPLGQMLNSLRKPI	3386
             :** :: :  :                              .:. .*:**::: *   :
```

```
hHUWE1      IRRSSLLTEKLLRLLSLISIALPENKVSEAQANSGSGASSTTT--ATSTTST-TTTTAAS	3499
mHUWE1      IRRSSLLTEKLLRLLSLISIALPENKVSEVQTNSSNSGSSTAATSNTSTTTT-TTTTATA	3503
zHUWE1      IRRSSLLTEKLLRLLSLISIALPDNKATEVPASHPVPQTPTATPSAP-------------	3654
dHUWE1      ICRSPHLTDMLVKLLASLSTELPKEEELLPLSDQM----PPLVPIGPVQRAGAAAAAAHP	4244
cEEL-1      MAKSPLK-EKVLKVASQIMVTLPMDTLKLLGNDE--------------------------	3419
            : :*    : :::: : :   ** :       .
```

```
hHUWE1      TTPTPPTAPTPVTSAPALVAATAISTIVVAASTTVTTPTTATTTVS--I-----SPTTKG	3552
mHUWE1      PTPTPPAATTPVTSAPALVAATAISTITVAASTTVTTPTTATTTVS--T------STTKG	3555
zHUWE1      -APQTTAGSGTI----SAVAATGTTQGSV--GSVGTTPSQPAATISVVAGSTTASSIIAG	3707
dHUWE1      PPPQPPAENTATA--------TEIGDGLAANIEEQASPA---------SGNPASGDSNAG	4287
cEEL-1      ------------------------------------------------------------	3419
```

```
hHUWE1      SKS---PAKVSDGGSSSTDFKMVSSGLTENQLQLSVEVLTSHSCSEEGLEDAANVLLQLS	3609
mHUWE1      SKS---PAKVGEGGS-GIDFKMVSSGLTENQLQLSVEVLTSHSCSEEGLEDAANVLLQLS	3611
zHUWE1      KLK---PA----VSITDIESKLASTGLTEKQLQLSVEVLTSHSCSEEGLEDAANILLQLS	3760
dHUWE1      AKRTQLPTLSVALPVTPSKPRRKIYSKNFSQLQLVIEVLTHQCGTTEGLDNVAKLIVNLT	4347
cEEL-1      -----------------------SRKPLAEKLEFVIQVMTTGSCSTEGLADGLTILSEAM	3456
                                         .:*:: ::*:*  . : *** :  .:: :
```

**G3639V**

```
hHUWE1      RGDSG-TRDTVLKLLLNGARHLGYTLCKQIGTLLAELREYNLEQQRRAQCETLSP-DGL-	3666
mHUWE1      RGDSG-TRDTVLKLLLNGARHLGYTLCKQIGTLLAELREYNLEQQRRAQCETLSP-DGL-	3668
zHUWE1      RGDAP-TRDTVLRLLLSGARHLGYTLCKQIGTLLAELREYNLEQQRRARADAQSP-EAS-	3817
dHUWE1      QCSQA-SSAIFIQYLTAAILGLAEEVRQAIQMLLNEIRMYNIHQGGAGTTSQAASTSGAA	4406
cEEL-1      RSLSDSTSVDIYEHLFLAVSKLGGELLPQVDRLIVELDEAQKKEEVSSVAPSDQPTSSK-	3515
            :     :   . . *  .   *.  :   :  *: *:   : .:   .        ..
```

```
hHUWE1      ------------PEEQPQTTKLKGKMQSRFDMAENVVIVASQ---KRPLGGRELQLPSMS	3711
mHUWE1      ------------PEEQPQTTKLKGKMQSRFDMAENVVIVASQ---KRPLGGRELQLPSMS	3713
zHUWE1      ------------HDDPSITTRLKGKMTSRFDGSESVVIVAAQ---KRTLGGRELQLPCMS	3862
dHUWE1      AATASYVPTVSGASLLPNLAVHEGIMQDRFTAE--HVIISAP---KNAKPTCELQLPSMK	4461
cEEL-1      --T---------AQLVVDTSAAGRVATGRFDGERLV-IDGDQNMRLQMSSCKELQLPAVT	3563
                         .     :       .**       * .      .     *****.:.
```

**T3717A**

```
hHUWE1      MLTSKTSTQKFFLRVLQVIIQLRDDTRRANKKAKQT-GRLGSSGLGSASSIQAAVRQLEA	3770
mHUWE1      MLTSKTSTQKFFLRVLQVIIQLRDDTRRANKKAKQT-GRLGSSGLGSASSIQAAVRQLEA	3772
zHUWE1      SLTSKTSTQKFFLRVLQVIIQLREDTRRANKKAKQTGGRLSSTSLGSASSIQAAVRQLEA	3922
dHUWE1      KLMSNSSAQPYFLRTLKIFMQVRDMYEAQNGLS--TGGGVACG-AG-------------D	4505
cEEL-1      VLTDKGGAQYALLSALQTLVKVRNHMKAIRKDKAKRAKD---------------------	3602
             * .: .:*  :* .*: ::::*:  .  .
```

```
hHUWE1      EADAIIQMVREGQRARRQQQAATSES-SQS------------------------------	3799
mHUWE1      EADAIIQMVREGQRARRQQQAATSESSNQS------------------------------	3802
zHUWE1      EADAIIQMVREGQRARRLQQAPPPSSSASSSAAAAAASAAVAVAGSSAPSTSATNNPPAG	3982
dHUWE1      NDDDIIDIEGDGGASGGSSSD-------------------------MAPGSASTISP---	4537
cEEL-1      -------------AEKKLKEKETPSTSAPAA--------------PAAPATAPTA-P---	3631
                              ..
```

```
hHUWE1      --------------EASVRREESPMDVDQPS---PSAQDTQSI-ASDGTPQGEKEKEERP	3841
mHUWE1      --------------ETSVRREESPMDVDQPS---PSAQDTQSIVISDGTPQGEKEKEEKP	3845
zHUWE1      TASAATSEVHTVAEPPAAQRDDSPMDVDQPS---PLEQDQPTLGEE---SSSQQEQEERL	4036
dHUWE1      --------------PPPMETNQIAQDGDDDEEDDDEARSFPGVGNH-------AVASASK	4576
cEEL-1      -----------AAPDAPVENNEVPEPA--------------------------AELEEVN	3654
                              . ::                                  .
```

**V3883A**

```
hHUWE1      PELPLLS-EQLSLDELWDMLGECLKELEESHDQHAVLVLQPAVEAFFLVHATERESKPPV	3900
mHUWE1      PELPLLS-EQLSLDELWDMLGECLKELEESHDQHAVLVLQPAVEAFFLVHATERESKPPV	3904
zHUWE1      PDLPLLS-EQLLLDELWDMLGECLKELEESHDQHAVLVLQPAVEAFFLVHATERESKPPV	4095
dHUWE1      STKPTLS-QILCLDVLWNTLSECLVALEESKDEFAVLVLQPTVEAFFLIHASHRGASKKS	4635
cEEL-1      DEEPRISERLSSLESLWNSLSECLLRLGKASDPHAVLALQPAAEAFFLVHASQQNKLKAK	3714
               * :* .   *: **: *.***  * :: * .***.***:.*****:**:.:
```

```
hHUWE1      RDTRESQ--------------LAHIKDE------PPPLSPAPLTPATPS-----------	3929
mHUWE1      RDTRESQ--------------LAHIKDE------PPPLSPAPLTPATPS-----------	3933
zHUWE1      RDTRESQ--------------LSHIKDE------PPPLSPAPLTPATPS-----------	4124
dHUWE1      GRSFMPNAAGFGLRAAAGAGAIGGLGAAAPSAQEYSPFMDDVSSANTPSSASFDDEPRMQ	4695
cEEL-1      DAEAKRKES------------------------------QAASSSA--------------	3730
                  :                                    :
```

**Q3968P**

```
hHUWE1      ---SLDPFFSREPS---SMHISSSLPPDTQKFLRFAETHRTVLNQILRQSTTHL-ADGPF	3982
mHUWE1      ---SLDPFFSREPS---SMHISSSLPPDTQKFLRFAETHRTVLNQILRQSTTHL-ADGPF	3986
zHUWE1      ---SLDPFFSREPS---SMHISSNLPPDTQKFLRFAETHRTVLNQILRQSTTHL-ADGPF	4177
dHUWE1      DASTNTPLVTNPSTSTAMSAHEAQLRQDRQKFLQFAEKHRTVLNQILRQSPTHL-SDGPF	4754
cEEL-1      --------AAAVASSVSHDGLREDLDPDTAKLIEFAEKHRQVLNQALRQNNAVLSAGGPF	3782
                     :   :         .*  *  *::.***.** **** ***. : * :.***
```

Hotspot 3 start

**R3998C**

```
hHUWE1      AVLVDYIRVLDFDVKRKYFRQELERLDEG---LRKEDMAVHVRRDHVFEDSYRELHRKSP	4039
mHUWE1      AVLVDYIRVLDFDVKRKYFRQELERLDEG---LRKEDMAVHVRRDHVFEDSYRELHRKSP	4043
zHUWE1      AVLVDYIRILDFDVKRKYFRQELERLDEG---LRKEDMAVHVRRDHVFEDSYRELHRKSP	4234
dHUWE1      AVLVDHTRILDFDVKRKYFQTELERLDEG---IRREEHTVSVRRVTVFEDSFRVLYRLGP	4811
cEEL-1      AILTQFPKLLDFDVKRKYFRKELTKLEPSMPRYRRNDVSVQVSRNRVFSDSFRELFRLRP	3842
            *:*.:. ::**********: ** :*: .    *::: :* * *  **.**:* *.*  *
```

HECT start

**G4086D**

```
hHUWE1      EEMKNRLYIVFEGEEGQDAGGLLREWYMIISREMFNPMYALFRTSPGDRVTYTINPSSHC	4099
mHUWE1      EEMKNRLYIVFEGEEGQDAGGLLREWYMIISREMFNPMYALFRTSPGDRVTYTINPSSHC	4103
zHUWE1      EDMKNRLYIVFEGEEGQDAGGLLREWYMIISREMFNPMYALFRTSPGDRVTYTINPSSHC	4294
dHUWE1      EEWKNRFYIVFEDEEGQDAGGLLREWYVIISREIFNPMYALFCVSPGDRVTYMINPSSHA	4871
cEEL-1      SEWKNRFYIIFQGEEGQDAGGLLREWFSVITREIFNPNYALFITAPGDMVTYMINKASYI	3902
            .: ***:**:*:.*************: :*:**:*** **** .:*** *** ** :*:
```

```
hHUWE1      NPNHLSYFKFVGRIVAKAVYDNRLLECYFTRSFYKHILGKSVRYTDMESEDYHFYQGLVY	4159
mHUWE1      NPNHLSYFKFVGRIVAKAVYDNRLLECYFTRSFYKHILGKSVRYTDMESEDYHFYQGLVY	4163
zHUWE1      NPNHLSYFKFVGRVVAKAVYDNRLLECYFTRSFYKHILGKSVRYTDMESEDYPFFQGLVY	4354
dHUWE1      NPNHLSYFKFVGRVIAKAVHDNKLLECYFTRSFYKHILGKQVKHTDMESQDYEFYKGLDY	4931
cEEL-1      NPEHLDYFKFVGRLIAKSVFEHKYLDCYFTRAFYKHILNLPVRYQDLESEDPAFFKSLDF	3962
            **:**.*******::**:*.::: *:*****:******.  *:: *:**:*  *::.* :
```

**R4187C**

```
hHUWE1      LLENDVSTLGYDLTFSTEVQEFGVCEVRDLKPNGANILVTEENKKEYVHLVCQMRMTGAI	4219
mHUWE1      LLENDVSTLGYDLTFSTEVQEFGVCEVRDLKPNGANILVTEENKKEYVHLVCQMRMTGAI	4223
zHUWE1      LLENDVSTLGYELTFSTEVQEFGVCEVRDLKANGANILVTEENKKEYVHLVCQMKMTGAI	4414
dHUWE1      LMKNDISTLGYELTFSTEVQEFGVTQIRDLKPNGRDTAVTEENKFEYVQLVCQLKMSGSI	4991
cEEL-1      LLQNPIDDLALDLTFSTEVEEFGVRSVRDLKPNGRKIEVNDANKDEYVKLVCQMKMTGSI	4022
            *::* :. *. :*******:**** .:**** ** .  *.: ** ***:****::*:*:*
```

**D3068G**

```
hHUWE1      RKQLAAFLEGFYEIIPKRLISIFTEQELELLISGLPTIDIDDLKSNTEYHKYQSNSIQIQ	4279
mHUWE1      RKQLAAFLEGFYEIIPKRLISIFTEQELELLISGLPTIDIDDLKSNTEYHKYQSNSIQIQ	4283
zHUWE1      RKQLAAFLEGFYEIIPKRLISIFTEQELELLISGLPTIDIDDLKANTEYHKYQSSSIQIQ	4474
dHUWE1      RQQLDAFLEGFYDIIPKHLISIFNEQELELLISGLPDIDIEDLKANTEYHKYTSKSAQIQ	5051
cEEL-1      RKQLDAFLTGFYEIIPKDLISMFNEQELELLISGLPTVDIDDMAANTDYKGFQKTSTHIQ	4082
            *:** *** ***:**** ***:*.************ :**:*: :**:*: : ..* :**
```

**G4310R**

```
hHUWE1      WFWRALRSFDQADRAKFLQFVTGTSKVPLQGFAALEGMNGIQKFQIHRDDRSTDRLPSAH	4339
mHUWE1      WFWRALRSFDQADRAKFLQFVTGTSKVPLQGFAALEGMNGIQKFQIHRDDRSTDRLPSAH	4343
zHUWE1      WFWRALRSFDQADRAKFLQFVTGTSKVPLQGFAALEGMNGIQKFQIHRDDRSTDRLPSAH	4534
dHUWE1      WFWRALRSFDQADRAKFLQFVTGTSKVPLQGFGSLEGMNGIQKFQIHRDDRSTDRLPCAH	5111
cEEL-1      WFWRALRSFEKEDKAKFLQFVTGTSKVPLQGFASLEGMNGVQKFSIHMDSRGGDRLPAAH	4142
            *********:: *:******************.:******:***.** *.*. ****.**
```

```
hHUWE1      TCFNQLDLPAYESFEKLRHMLLLAIQECSEGFGLA	4374
mHUWE1      TCFNQLDLPAYESFEKLRHMLLLAIQECSEGFGLA	4378
zHUWE1      TCFNQLDLPAYESYEKLRHMLLLAIQECSEGFGLA	4569
dHUWE1      TCFNQLDLPMYKSYDKLRSCLLKAIHECSEGFGFA	5146
cEEL-1      TCFNQLDLPQYESYEKLRQSLLLAIRECTEGFGFA	4177
            ********* *:*::***  ** **:**:****:*
```
